# Automated Image Analysis Reveals Different Localization of Synaptic Gephyrin C4 Splice Variants

**DOI:** 10.1523/ENEURO.0102-22.2022

**Published:** 2023-01-03

**Authors:** Filip Liebsch, Fynn R. Eggersmann, Yvonne Merkler, Peter Kloppenburg, Günter Schwarz

**Affiliations:** 1 Institute of Biochemistry, Department of Chemistry, University of Cologne, Cologne 50674, Germany; 2 Biocenter, Institute for Zoology, and Cologne Excellence Cluster on Cellular Stress Responses in Aging-Associated Diseases (CECAD), University of Cologne, Cologne 50674, Germany; 3 Institute of Biochemistry, Department of Chemistry, Center for Molecular Medicine Cologne (CMMC), University of Cologne, Cologne 50674, Germany

**Keywords:** adeno-associated virus, automated image analysis, gephyrin, inhibitory synapses, splice variants

## Abstract

Postsynaptic scaffolding proteins function as central organization hubs, ensuring the synaptic localization of neurotransmitter receptors, trans-synaptic adhesion proteins, and signaling molecules. Gephyrin is the major postsynaptic scaffolding protein at glycinergic and a subset of GABAergic inhibitory synapses. In contrast to cells outside the CNS, where one gephyrin isoform is predominantly expressed, neurons express different splice variants. In this study, we characterized the expression and scaffolding of neuronal gephyrin isoforms differing in the inclusion of the C4 cassettes located in the central C-domain. In hippocampal and cortical neuronal populations, gephyrin P1, lacking additional cassettes, is the most abundantly expressed isoform. In addition, alternative splicing generated isoforms carrying predominantly C4a, and minor amounts of C4c or C4d cassettes. We detected no striking difference in C4 isoform expression between different neuron types and a single neuron can likely express all C4 isoforms. To avoid the cytosolic aggregates that are commonly observed upon exogenous gephyrin expression, we used adeno-associated virus (AAV)-mediated expression to analyze the scaffolding behavior of individual C4 isoforms in murine dissociated hippocampal glutamatergic neurons. While all isoforms showed similar clustering at GABAergic synapses, a thorough quantitative analysis revealed localization differences for the C4c isoform (also known as P2). Specifically, synaptic C4c isoform clusters showed a more distal dendritic localization and reduced occurrence at P1-predominating synapses. Additionally, inhibitory currents displayed faster decay kinetics in the presence of gephyrin C4c compared with P1. Therefore, inhibitory synapse heterogeneity may be influenced, at least in part, by mechanisms relating to C4 cassette splicing.

## Significance Statement

A neuron receives and integrates thousands of different synaptic inputs. At synapses of the same type, i.e., using the same neurotransmitter, subtle differences in their molecular composition could account for functional changes. Diverse synapse populations could contain different isoforms of the same protein, which are themselves generated by alternative splicing. In this work, we focused on the neuronal isoforms of gephyrin, which organizes structures at most inhibitory synapses. While overall similar properties of all isoforms were observed, an in-depth automated analysis revealed that the localization of clusters from one of the isoforms was different. Finally, this indicates that the alternative splicing of gephyrin could be one of the factors influencing the organization of postsynaptic proteins at diverse synapse populations.

## Introduction

The postsynaptic side of neuronal synapses contains scaffolding proteins that keep neurotransmitter receptors, trans-synaptic adhesion molecules and additional functional elements with precise stoichiometries in place (Sheng and Kim, 2011). Gephyrin (Gphn), initially co-purified with glycine receptors (GlyRs), is an important postsynaptic scaffolding protein at inhibitory glycinergic and GABAergic synapses (Prior et al., 1992; Fritschy et al., 2008). It is ubiquitously expressed and in addition to its neuronal role, the protein serves a more “ancient” metabolic function in molybdenum cofactor biosynthesis throughout the whole body (Feng et al., 1998; Stallmeyer et al., 1999). Both Gphn functions are essential for postnatal survival (Feng et al., 1998; Grosskreutz et al., 2003; O’Sullivan et al., 2016).

Gphn consists of an N-terminal G-domain that has the propensity to trimerize and a C-terminal E-domain with the ability to dimerize and directly interact with the cytosolic loop of the GlyR β-subunit (Schwarz et al., 2001; Schrader et al., 2004; Sola et al., 2004). The terminal domains are connected by the central C-domain, which provides structural flexibility (Belaidi and Schwarz, 2013) and regulates fine-tuning of postsynaptic clustering of glycine receptors and GABA_A_ receptors (GABA_A_Rs) via protein-protein interactions and posttranslational modifications (Tyagarajan et al., 2011, 2013; Sander et al., 2013; Dejanovic et al., 2014b).

**Figure 1.**
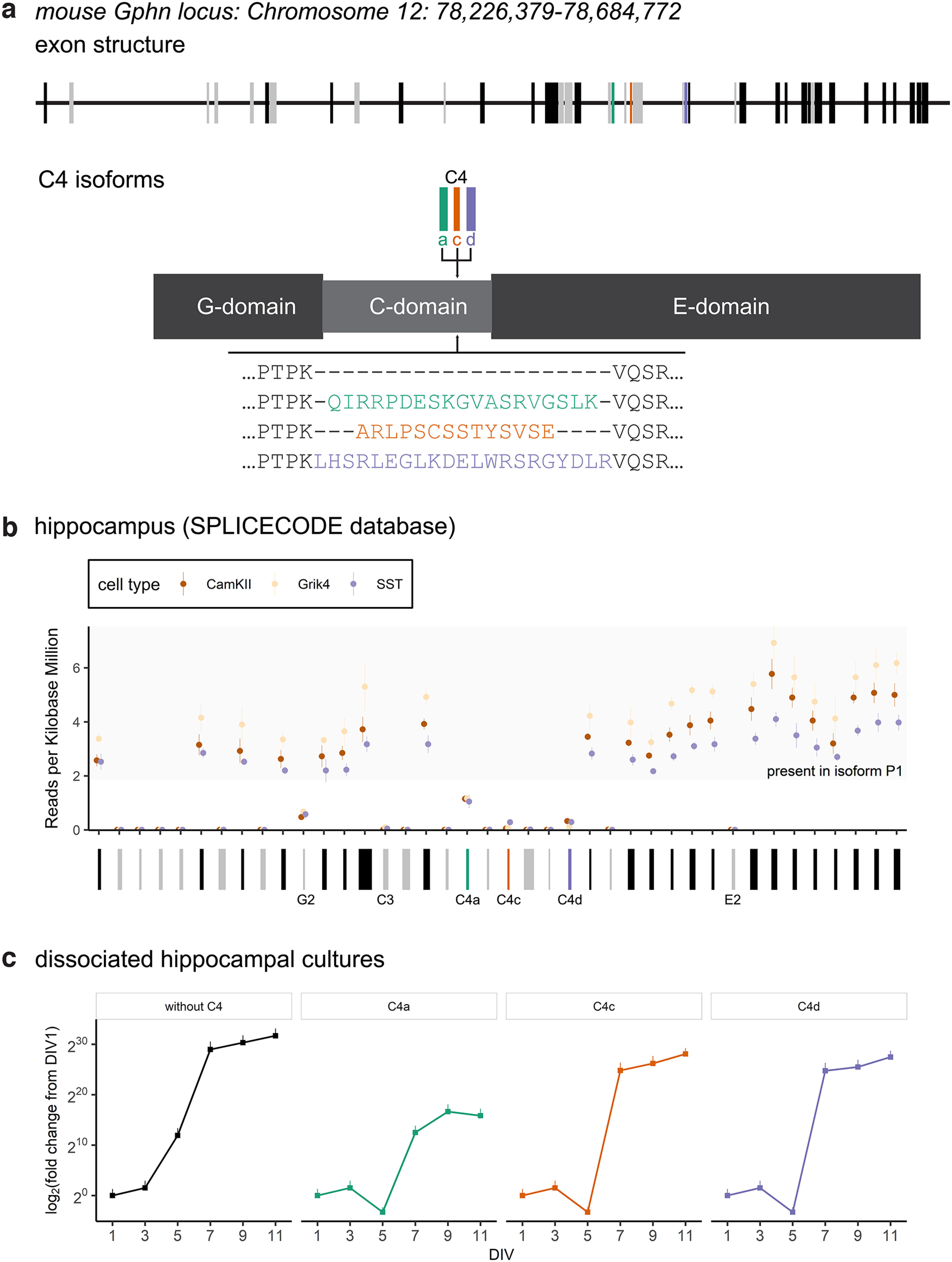
Alternative splicing of murine gephyrin. ***a***, The *Gphn* locus consists of 40 exons. Isoform P1 is generated from 22 exons (black) and the remaining 18 (gray, green, orange, purple) can contribute to numerous variants. Alternative splicing of exons in the C4 cluster results in the insertion of short sequences (14–21 aa, sequences from Rattus norvegicus are indicated here) in the C-domain, i.e., C4a (green), C4c (orange), or C4d (purple). ***b***, Normalized transcript expression (ribosome-associated mRNAs) in glutamatergic [Ca^2+^/calmodulin-dependent protein kinase II (CamKII) and glutamate receptor kainate type subunit 4 (Grik4)] and GABAergic [somatostatin (SST)] neurons, isolated from the hippocampus (SPLICECODE database). See Extended Data Figure 1-1. ***c***, Normalized transcript expression (housekeeping genes Rpl2 and β-actin) of C4 cassettes in dissociated hippocampal cultures during *in vitro* development, analyzed by quantitative PCR after 1, 3, 5, 7, 9, and 11 d *in vitro* (DIV; *N* = 3 independent cultures). Each data point represents the mean of all biological replicates, error bars represent standard deviation.

Since its discovery, multiple Gphn isoforms have been described in rodents and humans (Prior et al., 1992; Kirsch et al., 1993; Heck et al., 1997; Ramming et al., 2000; David-Watine, 2001; Rees et al., 2003). The murine gephyrin gene (*Gphn*) was originally described to consist of 29 exons (Heck et al., 1997; Meier et al., 2000; Ramming et al., 2000; Fritschy et al., 2008). A recent targeted approach, together with long-read sequencing, discovered 40 exons, and alternative splicing results in the expression of 277 unique transcripts (Dos Reis et al., 2022). The major gephyrin isoform in the brain is referred to as P1 and is generated from 22 exons (Prior et al., 1992; Fritschy et al., 2008; Dos Reis et al., 2022). The P1 open reading frame of human *GPHN* is 94% homologous to the rodent one, which translates to 99.7% amino acid conservation (Rees et al., 2003). All internal exons may undergo alternative splicing (Dos Reis et al., 2022) and traditionally certain exons were named according to the resulting alternative sequences in either of the three protein domains, e.g., G2, C3, C4a, C4c, C4d, and E2 (Fritschy et al., 2008; Fig. 1*a*).

10.1523/ENEURO.0102-22.2022.f1-1 Extended Data Figure 1-1 Alternative splicing of murine gephyrin in the cortex. Normalized transcript expression (ribosome-associated mRNAs) in glutamatergic [Ca2+/calmodulin-dependent protein kinase II (CamKII) and sodium channel epithelial 1 subunit α (Scnn1)] and GABAergic [somatostatin (SST), parvalbumin (PV), vasointestinal peptide (VIP)] neurons, isolated from the cortex (SPLICECODE database). Circle and range represent mean and SD. Download Figure 1-1, EPS file.

The inclusion of the G2 cassette results in a dominant-negative effect on GlyR clustering and prevents the enzyme’s ability to synthesize the molybdenum cofactor (Meier et al., 2000; Bedet et al., 2006; Smolinsky et al., 2008). The C3 cassette is mainly expressed in glial cells and tissues outside the brain; it is excluded from transcripts through NOVA proteins, neuronal regulators of pre-mRNA splicing (Ule et al., 2003; Licatalosi et al., 2008). Much less is known about the functional impact of C4 alternative splicing. Transcripts with C4 cassettes are predominantly found in neuronal tissue (Ramming et al., 2000; Rees et al., 2003; Smolinsky et al., 2008). The C4 cassettes can be present individually in single transcripts but are occasionally found in combination with other C4 cassettes within the same transcript (Ramming et al., 2000; Rees et al., 2003; Witte et al., 2019). In the first three postnatal weeks, exon skipping of the C4a cassette is increased, and this process is affected by the RNA-binding protein Sam68 (Witte et al., 2019). Additionally, different phosphorylation patterns were observed between P1 and C4c, when purified from insect cells (Herweg and Schwarz, 2012).

Here, we analyzed the neuronal expression and clustering of Gphn isoforms that differ in the inclusion of one of the C4 cassettes. We hypothesized that C4 isoforms could form structurally different scaffolds, which could in turn affect receptor clustering and/or synapse localization. Our results showed a relatively high abundance of C4a-containing transcripts in all major hippocampal and cortical neuron populations, while C4c and C4d were only detectable in low amounts. Interestingly, synaptic clustering in cultured neurons was similar for all C4 isoforms, suggesting that Gphn’s basic scaffolding properties remained unaltered following C4 splicing. However, an in-depth automated analysis of GABAergic synapses revealed that the addition of the C4c splice cassette resulted in more distally localized clusters. Additionally, when differentially labeled isoforms were co-expressed, we detected the largest effect between Gphn P1 and C4c variants, pointing to a unique role for the C4c-containing isoform. Functional experiments revealed that inhibitory currents displayed faster decay kinetics in the presence of gephyrin C4c compared with P1.

## Materials and Methods

### DNA constructs

Initially, pmEGFP-C2 was generated from pEGFP-C2 (Clonetech) by introducing the point mutation L221K through site-direted mutagenesis. Plasmids pAAV-hSyn-moxBFP and pAAV-hSyn-mEGFP were generated from moxBFP (a gift from Erik Snapp, Addgene plasmid #68064; http://n2t.net/addgene:68064; RRID:Addgene_68064) and pAAV-hSyn-mScarlet (a gift from Karl Deisseroth, Addgene plasmid #131001; http://n2t.net/addgene:131001; RRID:Addgene_131001) by PCR subcloning using BamHI and EcoRI. Next, pAAV-hSyn-moxBFP and pAAV-EF1a-mCherry-IRES-Flpo (a gift from Karl Deisseroth, Addgene plasmid #55634; http://n2t.net/addgene:55634; RRID:Addgene_55634) were used to generate pAAV-hSyn-moxBFP-IRES-Flpo using NEBuilder HiFi DNA Assembly (NEB). Finally, pAAV-CaMKIIa-EGFP (a gift from Bryan Roth, Addgene plasmid #50469; http://n2t.net/addgene:50469; RRID:Addgene_50469) and pAAV-hSyn-moxBFP-IRES-Flpo were used to generate pAAV-CaMKIIa-moxBFP-IRES-Flpo using NEBuilder HiFi DNA Assembly. From this template, the control pAAV-CaMKIIa-moxBFP was generated by deleting IRES-Flpo through site-directed mutagenesis.

Rat gephyrin C4c in pEGFP-C2 was previously described (Dejanovic et al., 2014a) and the gephyrin ORF was introduced into pAAV-hSyn-mScarlet or pAAV-hSyn-mEGFP by PCR subcloning using XcmI and SalI. The C4c cassette was either deleted or replaced with the C4a or C4d cassette using overlap extension PCR. These served as PCR templates to generate the inserts for Flp-dependent constructs. Plasmid backbones of pAAV-hSyn-mScarlet, excluding the mScarlet ORF and WPRE, were PCR amplified. FRT-sites and compatible overlapping ends were introduced into primers used for insert and plasmid backbone PCRs. Compatible overlapping ends were designed so that mScarlet-Gphn or mEGFP-Gphn ORFs were inverted on ligation using NEBuilder HiFi DNA Assembly.

The plasmids pAdDeltaF6 (Addgene plasmid #112867; http://n2t.net/addgene:112867; RRID:Addgene_112867) and pAAV2/1 (Addgene plasmid #112862; http://n2t.net/addgene:112862; RRID:Addgene_112862) were a gift from James M. Wilson.

All DNA constructs were confirmed by Sanger sequencing (Eurofins).

### Primary hippocampal cultures

Dissociated primary hippocampal cultures were prepared from C57BL/6NRj embryos (E17.5) of either sex. After dissociation, 65,000 cells were seeded on Poly-L-lysine coated 13-mm cover slips (#1) or 75,000 cells per well of a Poly-L-lysine coated 24-well plate. Cultures were grown in neurobasal medium supplemented with B-27, N-2, and L-glutamine (Thermo Fisher Scientific). Cultures were transfected after 8 d *in vitro* (DIV) with 400 ng plasmid DNA using Lipofectamine 2000 according to the manufacturer’s instructions. After 9 DIV, cultures were infected with a total of 2 × 10^8^ viral genome copies (GC). Recombinant AAV2/1 particles (1:1 Mix for co-infection experiments) were diluted in neurobasal medium supplemented with B-27, N-2, and L-glutamine (Thermo Fisher Scientific) before they were added to the cultures. To adjust the osmolality of the culture medium for electrophysiology experiments, 30 mm NaCl was added on DIV11.

### Transcript expression in primary hippocampal cultures

Cultures in 24-well plates were washed with PBS, harvested with a cell scraper, and resuspended in TRI reagent (Sigma). RNA was separated from other cellular components according to the manufacturer’s protocol and resuspended in 15 μl dH_2_O. In 10 μl, 400 ng RNA, 10 μm oligo (dT) primer, and 1 μm dNTPs were incubated at 65°C for 5 min and then chilled on ice. The RNA-mix was added to a 10 μl solution of RT buffer (Invitrogen), 25 mm MgCl_2_, 50 mm DTT and 1 μl SuperScript III RT (200 U/μl, Invitrogen). This mix was subjected to the following temperature cycle: 25°C 10 min, 50°C 60 min, 70°C 15 min, and subsequently chilled on ice. After incubation with 1 μl RNase A at 37°C for 20 min, the samples were stored at −20°C. For quantitative PCR (qPCR), 2 μl cDNA was added per well of a 384-well plate (Bio-Rad). A master mix, containing 2.5 μl ORA (qPCR Green ROX H Mix, 2× Master) mix and 0.5 μl of a 10 μm mixture of forward and reverse primer, was prepared and added to each sample. The plate was sealed with Microseal Adhesive Sealer (Bio-Rad) and spun down for 20 s at 300 × *g*. The plate was placed in a CFRX384 Real-Time System (Bio-Rad) and the qPCR was performed according to the ORA HighQ qPCR manual. Acquired cycle threshold (Ct) values were exported and normalized to the housekeeping control (RPL37a and Actinβ). ΔCt = Ct (gene of interest) – Ct (housekeeping gene). The fold change was calculated by dividing the 2^ΔCt^ by 2^ΔCt^ of the control condition.

### rAAV2/1 preparation

Recombinant AAV2/1 particles were prepared in HEK293T cells (DSMZ no. ACC 635) according to the detailed protocol by (Kimura et al., 2019). Viral particles were precipitated with PEG/NaCl and subsequently cleared via chloroform extraction (Kimura et al., 2019). Purity of viral preparations were assessed with SDS-PAGE and AAV titers determined using Gel green® (Biotium) protocol (Xu et al., 2020). Fluorescence was measured at 507 ± 5 nm excitation and 528 ± 5 nm emission using a plate reader equipped with monochromators (Tecan Spark).

### Immunocytochemistry

Cells were fixed with 4% formaldehyde in PBS for 15 min and quenched with NH_4_Cl in PBS at room temperature (RT). All subsequent incubation and wash steps (PBS) were performed at RT. After blocking/permeabilization for 1 h with 10% goat serum, 1% BSA, 0.2% Triton X-100 in PBS, the following primary antibodies (Synaptic Systems) were used: anti-vesicular GABA transporter (vGAT) (1:1000, #131003) for inhibitory presynaptic terminals; anti-GABA_A_R γ2 (1:500, #224004), anti-GABA_A_R α1 (1:500, #224204), and anti-GABA_A_R α2 (1:500, #224102) for postsynaptic GABA_A_Rs. The following secondary antibodies were used: goat anti-rabbit AlexaFluor 488 (1:500, #A-11034, Thermo Fisher Scientific), goat anti-rabbit AlexaFluor 647 (1:500, #A-21245, Thermo Fisher Scientific), and goat anti-guinea pig AlexaFluor 647 secondary antibodies (1:500, #ab150187, Abcam). Coverslips were mounted with Mowiol/Dabco.

### Confocal microscopy and image analysis

Image stacks [0.3-μm z-step size, 3432 × 3432 (144.77 × 144.77 μm)] were acquired on a Leica TCS SP8 LIGHTNING upright confocal microscope with an HC PL APO CS2 63×/1.30 glycerol objective, equipped with hybrid detectors (Leica HyD) and the following diode lasers: 405, 488, 552, and 638 nm. LIGHTNING adaptive deconvolution using “Mowiol” setting were applied. This form of adaptive image reconstruction is capable of theoretical resolutions down to 120 nm (lateral) and 200 nm (axial).

Images were segmented and analyzed in an automated fashion using ImageJ/FIJI 1.53c (https://imagej.net/software/fiji/) and the code is freely available online at https://github.com/FilLieb/quantitative_synapse_analysis. Maximum Z projections were used for image segmentation. Rolling ball background subtraction, median filtering, and auto thresholding methods were used: Default (for vGAT and GABA_A_Rɣ2 masks), Otsu (for soma, GABA_A_R α1, and GABA_A_R α2 masks), and mean (for neuron mask). Gephyrin cluster masks were generated by thresholding with local maxima identification using the ImageJ/FIJI find maxima and segmented particles function (Moreno Manrique et al., 2021). Individual regions of interest were classified according to their overlap with foreground in segmented masks and filtered with appropriate size ranges. For intensity measurements, mean background intensity (as defined by the respective masks) was subtracted from average Z projections.

### Electrophysiology

Whole-cell patch-clamp recordings under current clamp and voltage clamp were conducted on primary cultures of murine hippocampal neurons between DIV12 and DIV15 at 30°C. Neurons were visualized with a fixed-stage upright microscope (Axio Examiner.D1, Carl Zeiss GmbH) using a water-immersion objective (Plan-Apochromat, 40×, 1 numerical aperture, 2.5 mm working distance, Carl Zeiss GmbH). The microscope was equipped with fluorescence and infrared differential interference contrast optics (Dodt and Zieglgänsberger, 1994). Red fluorescing gephyrin-positive cells were visualized with an X-Cite 120 illumination system (EXFO Photonic Solutions). Recordings were performed with an EPC9 patch-clamp amplifier (HEKA) controlled by the program PatchMaster (version 2 × 90.5, HEKA running on Windows 10). Data were recorded using a Micro1401 data acquisition interface (Cambridge Electronic Design Limited) and Spike 2 (version 7.08, Cambridge Electronic Design Limited). Data were sampled at 20 kHz and low-pass filtered at 10 kHz with a four-pole Bessel filter. Offline, an FIR digital filter at 2 kHz was applied in Spike 2. Electrodes with tip resistances between 4 and 6 MΩ were made from borosilicate glass (0.86-mm inner diameter; 1.5-mm outer diameter; GB150-8P, Science Products) with a vertical pipette puller (PP-830, Narishige).

During the recording, neurons were continuously superfused at a flow rate of ∼2.5 ml min^−1^ with a carbonated (95% O_2_ and 5% CO_2_) extracellular saline containing (in mm): 125 NaCl, 21 NaHCO_3_, 2.5 KCl, 2 MgCl_2_, 1.2 NaH_2_PO_4_, 10 HEPES, 5 glucose, adjusted to pH 7.2 with NaOH. For the voltage clamp recordings, glutamatergic input was blocked with 5 × 10^−5^ m DL-2-amino-5-phosphonopentanoic acid (DL-AP5; BN0086, Biotrend Chemikalien GmbH) and 10^−5^ m 6-cyano-7-nitroquinoxaline-2,3-dione (CNQX; C127, Sigma-Aldrich). Na^+^ mediated action potentials were blocked by 10^−6^ m tetrodotoxin (TTX; T-550, Alomone, Jerusalem BioPark). Current-clamp recordings were performed with a pipette solution containing (in mm): 140 K-gluconate, 10 KCl, 2 MgCl_2_, 10 HEPES, 0.1 EGTA, adjusted to pH 7.2 with KOH. For voltage clamp recordings of miniature IPSCs (mIPSCs), the patch pipette solution contained (in mm): 133 CsCl, 2 MgCl_2_, 1 CaCl_2_, 10 HEPES, 10 EGTA, adjusted with CsOH to pH 7.2. The liquid junction potential between intracellular and extracellular solution for current-clamp (14.6 mV) and voltage-clamp (6.3 mV) recordings were calculated using the LJPcalc software (https://swharden.com/LJPcalc; arXiv:1403.3640v2; https://doi.org/10.48550/arXiv.1403.3640) and compensated accordingly.

Neurons were voltage clamped at −50 mV. Because of the high intracellular chloride concentration, GABA_A_ receptor-mediated currents were detected as inward currents. Data were analyzed at 50 s intervals after the recording stabilized (∼10 min after obtaining whole-cell configuration). Data analysis was performed with Spike 2 (version 7.08, Cambridge Electronic Design Limited), Igor Pro 6 (version 6.37, Wavemetrics), and GraphPad Prism (version 6.01, GraphPad Software Inc.). mIPSC frequency, amplitude, and decay were determined offline. Events in voltage clamp traces were automatically detected by the Minhee Analysis program (version 1.1.3; Kim et al., 2021), when the signal crossed a threshold that was set and adjusted manually depending on the noise level of the signal. Events were classified as mIPSCs when the calculated coefficient of determination *r*^2^ > 0.3 and tau 1 < τ < 300 ms. This semi-automated detection procedure was verified by visual inspection. Statistical analysis between the two splice variants was performed using a two-sided permutation test.

### Statistical analysis and data visualization

Individual data points, mean, confidence intervals (CIs), and standard deviation are displayed in the figures. Estimation statistics and plots were generated with the R package DABESTR (Ho et al., 2019). Data were tested for normality, using violation of the Shapiro–Wilk test at *p* < 0.01 as the criterion. Data were analyzed for homoscedasticity, using violation of Levene’s test at *p* < 0.01. Datasets not meeting normality or homoscedasticity assumptions were analyzed using nonparametric tests (as indicated in the figure legends). All other data were analyzed with the indicated parametric tests. For imaging experiments, *N* = 15 cells per condition from three independent cultures were analyzed. For electrophysiology experiments, *N* = 12 for P1 and *N* = 13 for C4c cells from five independent cultures were analyzed.

Statistical analyses and data visualization were performed using R version 4.1.0 (2021–05-18), lsmeans v. 2.30–0, emmeans v. 1.7.2, MASS v. 7.3–54, rstatix v. 0.7.0, ggpubr v. 0.4.0, svglite v2.0.0, RcolorBrewer v. 1.1–2, ggrepel v. 0.9.1, forcats v. 0.5.1, stringr v. 1.4.0, dplyr v. 1.0.7, purrr v. 0.3.4, readr v. 2.0.0, tidyr v. 1.1.3, tibble v. 3.1.3, ggplot2v. 3.3.5, tidyverse v. 1.3.1, rio v. 0.5.27, pacman v. 0.5.1, dabestr v.0.3.0 in Rstudio environment (https://www.rstudio.com/), Platform: x86 64-w64-mingw32/x64 (64-bit). Figures were prepared with OMERO (https://www.openmicroscopy.org/) and Inkscape (https://inkscape.org/).

### Ethics statement

We complied with all relevant ethical regulations for animal testing and research. Experiments were approved by the local research ethics committees (Germany, Landesamt für Natur, Umwelt und Verbraucherschutz Nordrhein-Westfalen, reference 2016.A466 and 2021.A450).

### Data availability

Plasmids will be made available via Addgene (https://www.addgene.org/): pAAV-hSyn-fDIO-mScarlet-Gphn_P1 (Addgene plasmid # 194972), pAAV-hSyn-fDIO-mScarlet-Gphn_C4a (Addgene plasmid # 194973), pAAV-hSyn-fDIO-mScarlet-Gphn_C4c (Addgene plasmid # 194974), pAAV-hSyn-fDIO-mScarlet-Gphn_C4d (Addgene plasmid # 194975), pAAV-hSyn-fDIO-mEGFP-Gphn_P1 (Addgene plasmid # 194978), pAAV-CamKIIa-moxBFP-WPRE (Addgene plasmid # 194976), pAAV-CamKIIa-moxBFP-IRES-Flpo (Addgene plasmid # 194977).

### Code accessibility

The code for automated image analysis written in ImageJ Macro Language is freely available online at https://github.com/FilLieb/quantitative_synapse_analysis. The code is available as Extended Data 1. The code was run under Windows 10 × 64 (Build 19 042) on an HP Pavilion Desktop PC570-p0xx.

10.1523/ENEURO.0102-22.2022.ed1 Extended Data 1 The code for automated image analysis. Download Extended Data 1, ZIP file

## Results

### Neuronal expression of alternatively spliced Gphn transcripts

To analyze whether the sequences encoded by *Gphn* exons are part of translated transcripts (ribosome-associated mRNAs) in neurons, we retrieved their normalized expression (reads per kilobase million) from the SPLICECODE database (Furlanis et al., 2019). In hippocampal glutamatergic [Ca^2+^/calmodulin-dependent protein kinase II (CamKII) and glutamate receptor kainate type subunit 4 (Grik4)] and GABAergic [somatostatin (SST)] neurons, transcript expression of 27 exons was detected (Fig. 1*b*). High reads per kilobase million were detected for the 22 exons coding for the *Gphn* isoform P1. The expression of E2 and any of the recently discovered exons (Dos Reis et al., 2022) was below the limit of detection in the dataset. The expression profile in cortical [glutamatergic; CamKII (sodium channel epithelial 1 subunit α; Scnn1a), Grik4; and GABAergic (SST, parvalbumin; Pvalb, vasointestinal peptide; Vip)] neurons was comparable to the cells in the hippocampus (Extended Data Fig. 1-1).

By calculating the average reads per kilobase million for all cell types, we estimated that ∼64% of all transcripts in hippocampal neurons encode for the isoform P1 (59% in cortical neurons). Consequently, the remainder of transcripts consisted of 10% G2 (9% in cortex), 1% C3 (1% in cortex), 19% C4a (21% in cortex), 2% C4c (4% in cortex), and 4% C4d (6% in cortex). Overall, these data suggest that in the major neuron types in the murine hippocampus and cortex *in vivo* (Furlanis et al., 2019), most *Gphn* mRNAs code for P1 and predominantly five cassette exons can be used to generate alternatively spliced transcripts. It is important to notice that mRNA abundance correlates only partially with protein levels (Vogel and Marcotte, 2012; Smith et al., 2013; Liu et al., 2016; Aebersold et al., 2018).

The dominant-negative function of Gphn G2 (Bedet et al., 2006; Smolinsky et al., 2008) and reduced oligomerization as well as lower receptor affinity of the predominantly non-neuronally expressed isoform of Gphn C3 have been previously studied (Herweg and Schwarz, 2012; Nawrotzki et al., 2012). To gain insight into the properties of the thus far little explored Gphn C4 isoforms, we undertook a reductionist approach and analyzed expression and clustering of C4 isoforms in dissociated primary hippocampal cultures. The endogenous expression of transcripts coding for the different C4 isoforms increases over time in hippocampal cultures (Fig. 1*c*). While transcript levels without C4 increase after 5 d *in vitro* (DIV), those containing C4a, C4c, or C4d increase after 7 DIV. After 9 and 11 DIV, all transcript levels plateaued (Fig. 1*c*).

Overall, in agreement with previous work, Gphn C4 isoforms are expressed in major neuronal populations in hippocampus and cortex. In dissociated neuronal cultures, levels of Gphn transcripts with and without C4 cassettes increased during times of synaptogenesis (Deng et al., 2007).

### Exogenous Gphn C4 isoforms localize to GABAergic synapses

Recombinantly expressed Gphn in non-neuronal and neuronal cells has the tendency to form cytosolic aggregates, in the field often referred to as “blobs” (Kirsch et al., 1995; Schrader et al., 2004; Saiyed et al., 2007). To characterize potential differences between Gphn C4 isoforms in the absence of abnormal cytosolic aggregates, we developed a system to express low amounts of fluorophore-tagged Gphn in individual glutamatergic hippocampal neurons. Recombinant adeno-associated viruses (rAAVs) encapsidate single-stranded DNA and require the slow process of complementary-strand synthesis for transgene expression (McCarty et al., 2001). Therefore, we expected that cytosolic Gphn aggregation could be minimized by using rAAV-mediated infection.

**Figure 2.**
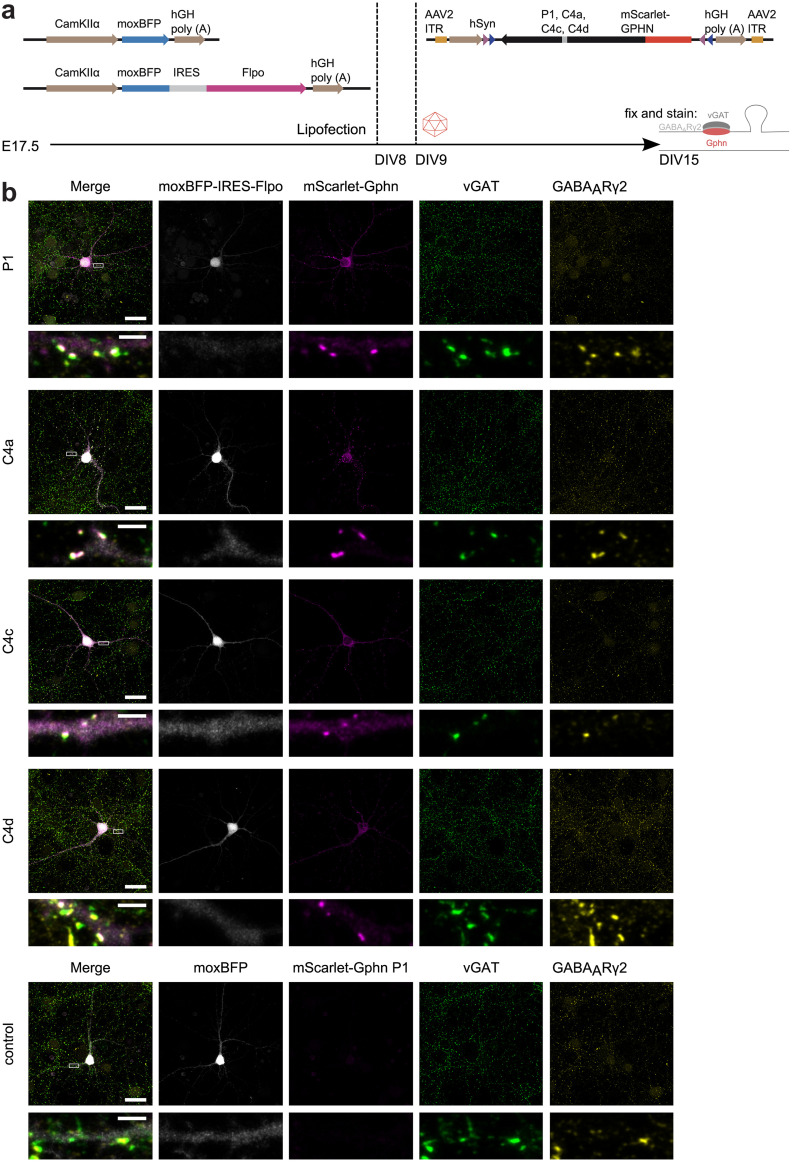
Expression of mScarlet-tagged Gphn C4 isoforms in CamKIIα-expressing murine hippocampal neurons. ***a***, Hippocampal cultures were transfected after 8 DIV with moxBFP or moxBFP-IRES-Flpo under the control of the CamKIIα promoter. After 9 DIV, cultures were infected with adeno-associated virus (AAV) 2/1 carrying Flp dependent mScarlet-tagged Gphn C4 variants as transgene. Cells were immunostained for inhibitory presynaptic terminals (vGAT) and postsynaptic GABA_A_Rɣ2 after 15 DIV. ***b***, Representative confocal images (with adaptive image reconstruction) of neurons expressing moxBFP-IRES-Flpo (or moxBFP as control) and mScarlet-tagged Gphn C4 isoforms. Scale bars: 25 μm and 2.5 μm in insets.

For single-cell analysis, dissociated hippocampal cultures were transfected with the recombinase Flpo and a cytosolic fluorescent protein (monomeric “ox” blue fluorescent protein, moxBFP) to fill the entire cell. The expression of this construct was restricted to glutamatergic cells through the use of the CamKIIα promoter (Wang et al., 2013). Next, Flp-dependent mScarlet-tagged Gphn was transduced using recombinant AAV 2/1 particles and immunocytochemistry revealed inhibitory presynaptic terminals and postsynaptic receptors (vGAT and GABA_A_Rɣ2, respectively; Fig. 2*a*). As expected, mScarlet signals were only detected in moxBFP-IRES-Flpo cells but were absent from cells transfected with moxBFP alone. Only occasionally, we observed few and small cytosolic exogenous mScarlet-Gphn aggregates or “blobs” (Fig. 2*b*). All Gphn C4 isoforms showed a clustered expression and regularly colocalized with vGAT and GABA_A_Rɣ2 clusters.

**Figure 3.**
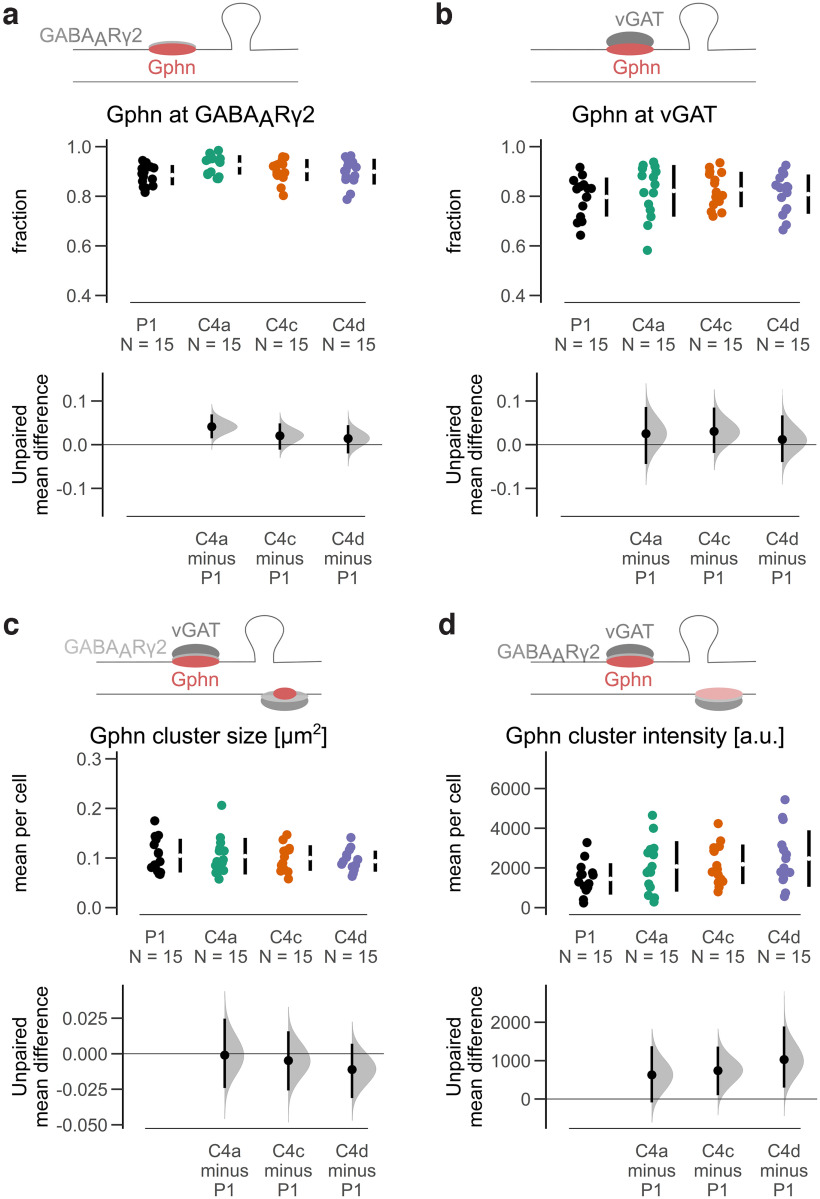
Quantitative analysis of mScarlet-tagged Gphn C4 isoform clusters. ***a***, The fraction of GABA_A_Rɣ2-positive clusters is unchanged between isoforms. ANOVA *F*_(3,56)_ = 2.243, *p* = 0.093. ***b***, The fraction of vGAT-positive clusters is indistinguishable between isoforms. ANOVA *F*_(3,56)_ = 0.395, *p* = 0.757. ***c***, Gphn cluster sizes (average per cell) of synaptic (vGAT-positive and GABA_A_Rɣ2-positive) clusters are not different between isoforms. ANOVA *F*_(3,56)_ = 0.432, *p* = 0.738. ***d***, Gphn cluster fluorescence intensities (average per cell) of synaptic (vGAT-positive and GABA_A_Rɣ2-positive) clusters are similar between isoforms. ANOVA *F*_(3,56)_ = 2.134, *p* = 0.106. Each data point represents the mean for an individual neuron. The filled curves indicate the resampled Δ distribution (5000 bootstrap samples) derived from the observed data. The Δ is indicated by the black circle. The 95% confidence interval of the mean difference is illustrated by the black vertical line. See Extended Data Figure 3-1.

To analyze Gphn C4 isoform clustering, we developed an automated and high-content image processing pipeline for image segmentation, intensity measurements, and localization. A total of 11 319 clusters (P1: 2777; C4a: 3187; C4c 2857; C4d: 2498) from 60 cells (15 per condition from three independent cultures) were detected and analyzed for various parameters. All C4 isoforms showed an average colocalization with GABA_A_Rɣ2 clusters of ∼90% and ∼80% with vGAT clusters. No differences between the isoforms were observed (Fig. 3*a*,*b*). Furthermore, neither the size, nor the fluorescence intensity (which is proportional to the number of Gphn molecules) of inhibitory synaptic (vGAT-positive and GABA_A_Rɣ2-positive) clusters were different among the C4 isoforms (Fig. 3*c*,*d*; Extended Data Fig. 3-1*a*). Additionally, similar numbers of synaptic Gphn clusters per cell area were observed for all C4 isoforms (Extended Data Fig. 3-1*b*).

10.1523/ENEURO.0102-22.2022.f3-1 Extended Data Figure 3-1 Quantitative analysis of synaptic mScarlet-Gphn C4 isoform clusters. ***a***, Cumulative frequency of size for all detected synaptic (vGAT-positive and GABA_A_Rɣ2-positive) Gphn clusters. ***b***, Number (#) of synaptic (vGAT-positive and GABA_A_Rɣ2-positive) Gphn clusters per cell are similar between isoforms; Kruskal–Wallis test, H(3) = 7.17, *p* = 0.067. Each data point represents the mean for an individual neuron. The filled curves indicate the resampled Δ distribution (5.000 bootstrap samples) derived from the observed data. The Δ is indicated by the black circle. The 95% confidence interval of the mean difference is illustrated by the black vertical line. Download Figure 3-1, EPS file.

**Figure 4.**
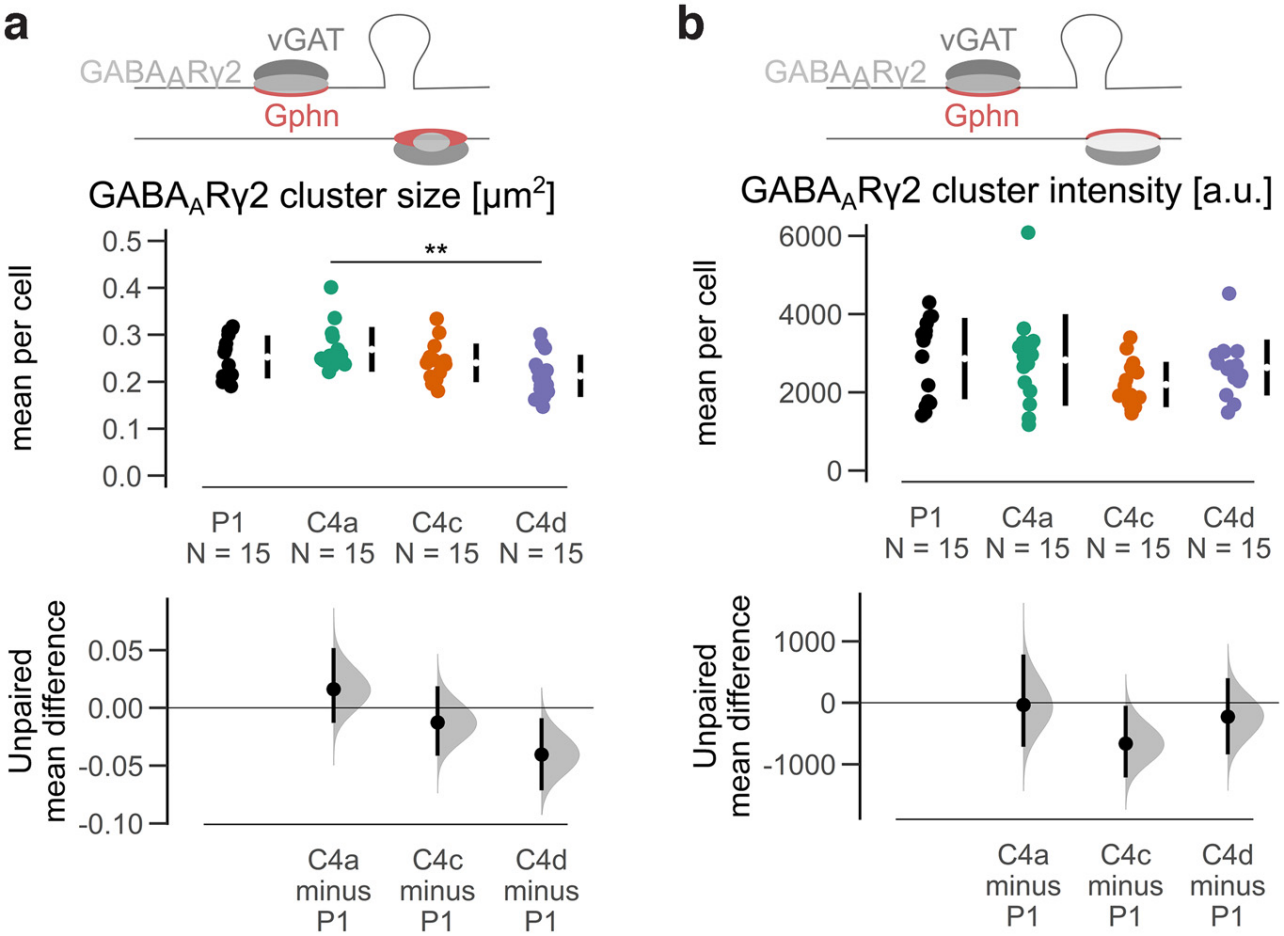
Quantitative analysis of GABA_A_Rɣ2 at mScarlet-tagged Gphn C4 isoform clusters. ***a***, GABA_A_Rɣ2 cluster sizes (average per cell) of synaptic (vGAT-positive) clusters are larger at C4a compared with C4d, while all other comparisons are not different between isoforms. Kruskal–Wallis test, H(3) = 11.18, *p* = 0.011, pairwise Wilcoxon rank test (Bonferroni corrected); **p < 0.01. ***b***, GABA_A_Rɣ2 cluster fluorescence intensities (average per cell) of synaptic (vGAT-positive) clusters are similar between isoforms. ANOVA *F*_(3,56)_ = 1.687, *p* = 0.180. Each data point represents the mean for an individual neuron. The filled curves indicate the resampled Δ distribution (5000 bootstrap samples) derived from the observed data. The Δ is indicated by the black circle. The 95% confidence interval of the mean difference is illustrated by the black vertical line. See Extended Data Figure 4-1.

Next, we analyzed whether the expression of Gphn C4 isoforms affected GABA_A_R clustering. When we selected synaptic GABA_A_Rɣ2 clusters (vGAT-positive), larger clusters were observed at Gphn C4a compared with Gphn C4d, while all other comparisons did not reveal differences (Fig. 4*a*; Extended Data Fig. 4-1*a*). Additionally, no differences in GABA_A_Rɣ2 cluster intensities at the different C4 isoform clusters were observed (Fig. 4*b*; Extended Data Fig. 4-1*b*). Importantly, the cluster size and cluster intensities of synaptic GABA_A_Rɣ2 clusters was increased at all exogenously expressed C4 isoforms compared with endogenous clusters (Extended Data Fig. 4-1*c*,*d*).

10.1523/ENEURO.0102-22.2022.f4-1 Extended Data Figure 4-1 Quantitative analysis of GABA_A_Rɣ2 at mScarlet-tagged Gphn C4 isoform clusters. ***a***, Cumulative frequency of size for all detected synaptic (vGAT-positive) GABA_A_Rɣ2 clusters at mScarlet-Gphn C4 isoform clusters. ***b***, Cumulative frequency of average fluorescence intensity for all detected synaptic (vGAT-positive) GABAARɣ2 clusters at mScarlet-Gphn C4 isoform clusters. ***c***, Cumulative frequency of size for all detected synaptic (vGAT-positive) GABA_A_Rɣ2 clusters at mScarlet-Gphn positive or negative clusters. ***d***, Cumulative frequency of average fluorescence intensity for all detected synaptic (vGAT-positive) GABA_A_Rɣ2 clusters at mScarlet-Gphn positive or negative clusters. Download Figure 4-1, EPS file.

Altogether, the data show that the basic scaffolding properties and association with GABAergic synapses of C4 isoforms exhibited only minor changes by C4 cassette insertion in cultured hippocampal glutamatergic neurons. The size of GABA_A_Rɣ2 clusters at Gphn C4a were larger compared with clusters at C4d.

### Exogenous Gphn C4c clusters are more distally localized

**Figure 5.**
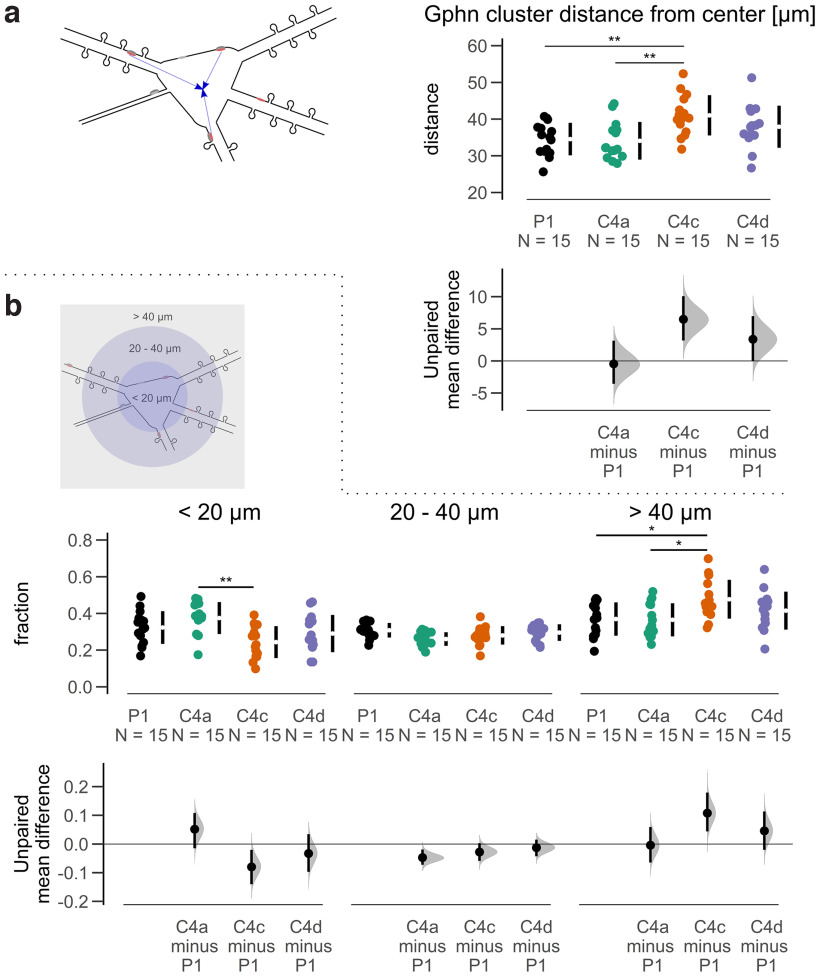
Quantitative analysis of mScarlet-tagged Gphn cluster distances. ***a***, Distances of synaptic (vGAT-positive) clusters from the center of the neuron’s soma are increased for isoform C4c compared with P1 and C4a ANOVA *F*_(3,56)_ = 5.783, *p* = 0.002, Tukey’s *post hoc* test; **p < 0.01. ***b***, Fraction of synaptic (vGAT-positive and GABA_A_Rɣ2-positive) clusters are increased for C4a versus C4c or C4d at a radius of 20 μm and increased for C4c versus P1 or C4a at a radius >40 μm around the neuron’s center. Data were analyzed with a two-way mixed ANOVA. There was a statistically significant two-way interaction between Gphn isoform and distance on fraction of synaptic clusters, *F*_(4,74)_ = 4.683, *p* = 0.002. Considering the Bonferroni adjusted *p*-values, the simple main effect of Gphn isoform was significant at <20 μm (*p* = 0.006) and >40 μm (*p* = 0.024) but not at 20–40 μm (*p* = 0.057). Pairwise comparisons show that the mean fraction of synaptic clusters was significantly different in C4a versus C4c (*p* = 0.0014) comparison at <20 μm; in P1 versus C4c (*p* = 0.0233) and in C4a versus C4c (*p* = 0.0168) at <40 μm. **p < 0.01, *p < 0.05. Each data point represents the mean for an individual neuron. The filled curves indicate the resampled Δ distribution (5000 bootstrap samples) derived from the observed data. The Δ is indicated by the black circle. The 95% confidence interval of the mean difference is illustrated by the black vertical line. See Extended Data Figure 5-1.

To investigate the cellular distribution of synaptic Gphn clusters (vGAT-positive), we measured their distances to the neuron’s center. We observed significantly larger distances for isoform C4c compared with P1 and C4a, while the difference between C4c and C4d was not significant (Fig. 5*a*). In agreement with this, larger distances were detected for GABA_A_Rɣ2 clusters localized at isoform C4c compared with P1 and C4a, while the difference between C4c and C4d was not statistically significant (Extended Data Fig. 5-1*a*).

10.1523/ENEURO.0102-22.2022.f5-1 Extended Data Figure 5-1 Quantitative analysis of cluster distances. ***a***, Distances of GABA_A_Rɣ2 (Gphn-positive) clusters from the center of the neuron’s soma are increased for isoform C4c compared with P1 and C4a *F*_(3,56)_ = 4.54, *p* = 0.006, Tukey’s *post hoc* test; *p < 0.05. ***b***, The fraction of synaptic (vGAT-positive and GABA_A_Rɣ2-positive) Gphn clusters are changed for C4a, C4c, and C4d. Data were analyzed with a two-way mixed ANOVA. There was a statistically significant two-way interaction between Gphn isoform and distance on fraction of synaptic clusters, *F*_(4,74)_ = 4.683, *p* = 0.002. Considering the Bonferroni adjusted *p*-values, the simple main effect of distance was significant at C4a (*p* < 0.0001), C4c (*p* < 0.001), and C4d (*p* = 0.0014) but not at P1 (*p* = 0.352). Pairwise comparisons show that the mean fraction of synaptic clusters was significantly different in <20 versus 20–40 μm (*p* < 0.001) and 20–40 versus >40 μm (*p* = 0.0014) comparison at C4a; in <20 versus >40 μm (*p* < 0.001) and in 20–40 versus >40 μm (*p* < 0.001) at C4c; in <20 versus >40 μm (*p* = 0.0010) and in 20–40 versus >40 μm (*p* = 0.0017) at C4d. Each data point represents the mean for an individual neuron. The filled curves indicate the resampled Δ distribution (5.000 bootstrap samples) derived from the observed data. The Δ is indicated by the black circle. The 95% confidence interval of the mean difference is illustrated by the black vertical line. Download Figure 5-1, EPS file.

To characterize this effect in more detail, synaptic Gphn clusters were classified according to their distance from the neuron’s center: 20 μm radius (somatic and perisomatic clusters), 20 to 40 μm radius and >40 μm radius (distally located clusters). The analysis revealed that the fraction of synaptic clusters at these distances differed for the Gphn isoforms (mixed two-way ANOVA, *p* < 0.01). Specifically, the fraction of synaptic clusters was reduced for C4c versus C4a for the somatic and perisomatic clusters (*r* < 20 μm) and increased for C4c versus P1 and C4a for distally located clusters (*r* > 40 μm; Fig. 5*b*). When the effect was analyzed at each isoform, the fraction of synaptic P1 clusters showed no difference between the distances. Synaptic C4a clusters showed a higher fraction of perisomatic clusters (*r* < 20 μm) and distally located clusters (*r* > 40 μm) compared with the intermediate location (20–40 μm). Both synaptic C4c and C4d clusters showed a higher fraction of distally located clusters (*r* > 40 μm) compared with the other locations (Extended Data Fig. 5-1*b*).

Collectively, Gphn C4 isoforms localized differently in neurons. Here, the presence of a 14-residue sequence encoded by the C4c splice cassette in the C-domain resulted in clusters that were localized further away from the neuron’s center than clusters of P1 and C4a isoforms, while Gphn C4d displayed an intermediate behavior.

### Reduced localization of Gphn C4c at synaptic P1 clusters

**Figure 6.**
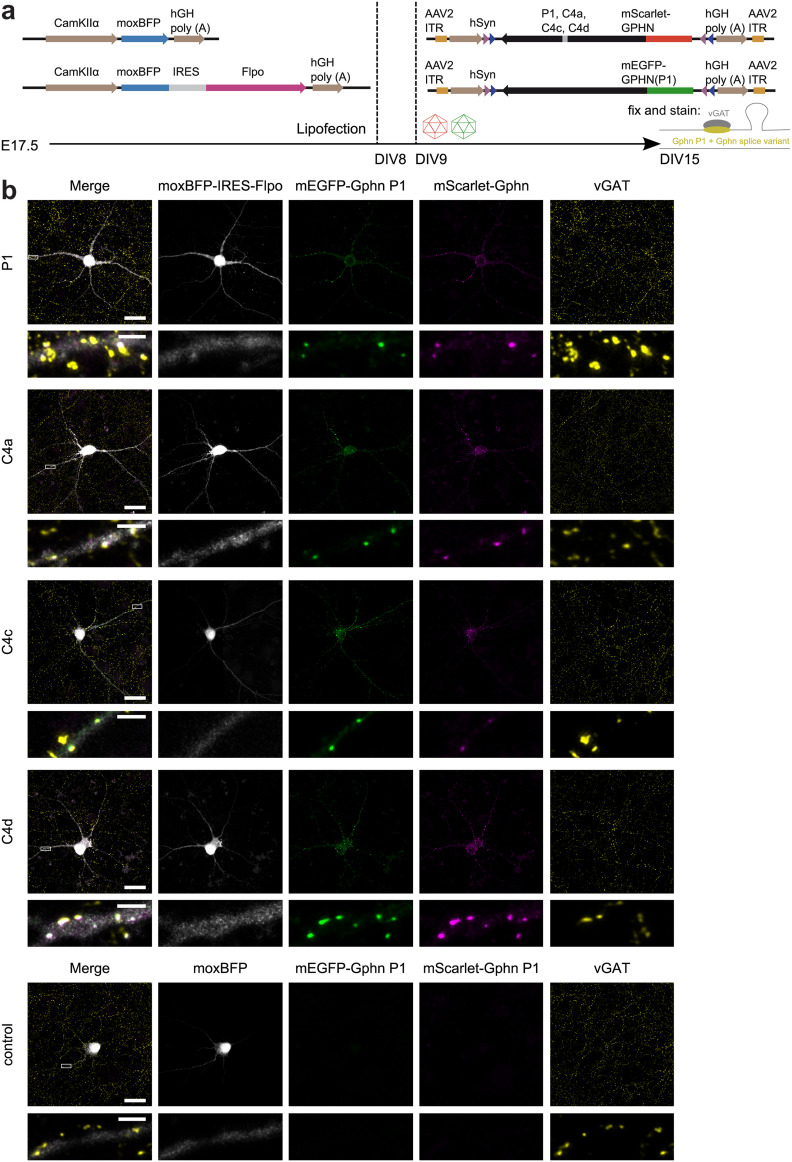
Co-expression of mEGFP-tagged Gphn P1 and mScarlet-tagged Gphn C4 isoforms in CamKIIα-expressing murine hippocampal neurons. ***a***, Hippocampal cultures were transfected after 8 DIV with moxBFP or moxBFP-IRES-Flpo under the control of the CamKIIα promoter. After 9 DIV, cultures were transduced with adeno-associated virus (AAV) 2/1 carrying Flp-dependent mEGFP-tagged Gphn P1 and mScarlet-tagged Gphn C4 variants as transgenes, respectively. ***b***, Representative confocal images (with adaptive image reconstruction) of neurons expressing moxBFP-IRES-Flpo (or moxBFP as control), mEGFP-tagged Gphn P1 and mScarlet-tagged Gphn C4 isoforms. Scale bars: 25 μm and 2.5 μm in insets.

We conducted our exogenous C4 isoform analyses in dissociated murine glutamatergic hippocampal neurons that express Gphn P1 as most abundant variant (Fig. 1*b*; Dos Reis et al., 2022). In order to directly compare the neuronal localization and scaffolding properties between P1 and C4 isoforms containing one of the C4 cassettes within the same neuron, we extended our imaging system by simultaneously expressing differently tagged Gphn P1 and Gphn C4 isoforms. With this setup, we assessed whether the introduction of sequences encoded by the individual C4 cassettes either change the properties of P1 or whether C4 isoforms are equally well recruited to P1 clusters. Analogous to our previous approach, single CamKIIɑ-expressing hippocampal neurons were filled with moxBFP and subsequently infected with AAV2/1 to express mEGFP-tagged Gphn P1 together with one mScarlet-tagged Gphn C4 isoform (Fig. 6*a*). As expected, mScarlet and mEGFP signals were only detected in moxBFP-IRES-Flpo cells and absent from moxBFP-transfected cells (Fig. 6*b*).

**Figure 7.**
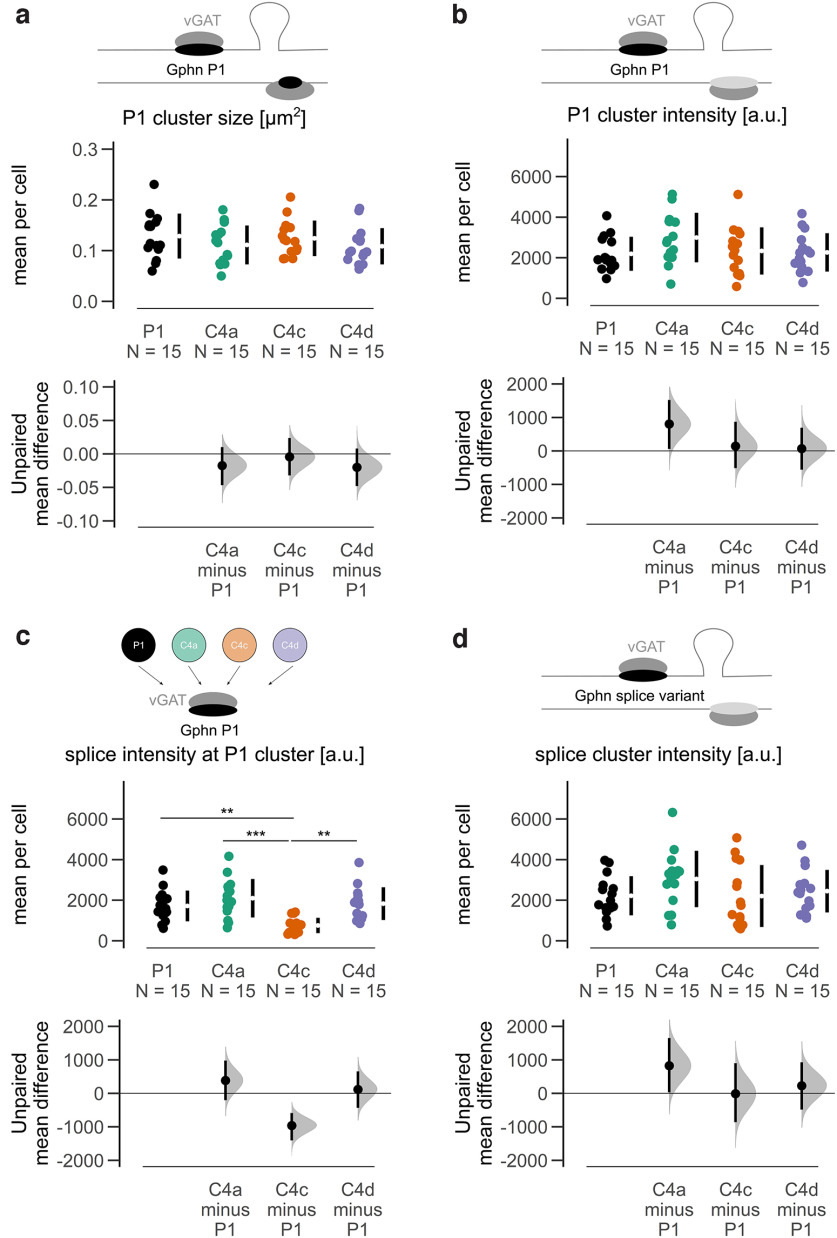
Quantitative analysis of simultaneously expressed mEGFP-tagged Gphn P1 and mScarlet-tagged Gphn C4 isoforms. ***a***, Cluster sizes (average per cell) of synaptic (vGAT-positive) P1 clusters are not affected by isoform co-expression. ANOVA *F*_(3,56)_ = 0.967, *p* = 0.415. ***b***, Cluster fluorescence intensities (average per cell) of synaptic (vGAT-positive) P1 clusters remain unchanged by isoform co-expression. ANOVA *F*_(3,56)_ = 1.856, *p* = 0.148. ***c***, Cluster fluorescence intensities (average per cell) of synaptic (vGAT-positive) Gphn C4c is reduced at P1 clusters compared with all other isoforms. ANOVA *F*_(3,56)_ = 9.099, *p* < 0.001, Tukey’s *post hoc* test; **p < 0.01, ***p < 0.001. ***d***, Cluster fluorescence intensities (average per cell) of synaptic (vGAT-positive) C4 isoform clusters remain unchanged by P1 co-expression. ANOVA *F*_(3,56)_ = 1.467, *p* = 0.233. Each data point represents the mean for an individual neuron. The filled curves indicate the resampled Δ distribution (5000 bootstrap samples) derived from the observed data. The Δ is indicated by the black circle. The 95% confidence interval of the mean difference is illustrated by the black vertical line. See Extended Data Figure 7-1.

To detect potential differences, we employed our automated imaging analyses. A total of 7026 (P1: 1287; C4a: 2017; C4c 1939; C4d: 1783) synaptic (vGAT-positive) mEGFP-tagged Gphn P1 clusters from 60 cells (15 per condition from three independent cultures) were inspected. First, we studied whether P1 clustering was affected by additional C4 isoform expression. Neither cluster size nor intensity of mEGFP-Gphn P1 was altered in the presence of mScarlet-Gphn P1, C4a, C4c, or C4d (Fig. 7*a*,*b*). Next, we quantified splice variant fluorescence intensity at synaptic P1 clusters. Here, we discovered that C4c intensity at P1 clusters was significantly and strongly reduced (ANOVA effect size, η^2^ = 0.3277) compared with all other conditions (Fig. 7*c*). This reduction in C4c fluorescence intensity at P1 clusters was not because of overall reduced cluster size or cluster intensity (Extended Data Fig. 7-1*a*; Fig. 7*d*) and is in agreement with the findings that Gphn C4c was enriched at distal synapses (Fig. 3*c*,*d*), showing that C4 cassette insertion did not affect cluster size and intensity when fluorescently labeled isoforms were expressed individually.

10.1523/ENEURO.0102-22.2022.f7-1 Extended Data Figure 7-1 Quantitative analysis of simultaneously expressed mEGFP-tagged Gphn P1 and mScarlet-tagged Gphn C4 isoforms. ***a***, Cluster sizes (average per cell) of synaptic (vGAT-positive) C4 isoform clusters remain unchanged upon P1 co-expression. *F*_(3,56)_ = 1.75, *p* = 0.167. Each data point represents the mean for an individual neuron. The filled curves indicate the resampled Δ distribution (5.000 bootstrap samples) derived from the observed data. The Δ is indicated by the black circle, which is horizontally aligned with the mean of the test group. The 95% confidence interval of the mean difference is illustrated by the black vertical line. ***b***, Individual fluorescence intensities at synaptic (vGAT-positive) Gphn P1 clusters. Data were analyzed with Spearman correlation. P1 ρ = 0.92 (95% CI [0.91–0.93]), *p* < 0.001; C4a ρ = 0.92 (95% CI [0.91–0.93]), *p* < 0.001; C4c ρ = 0.78 (95% CI [0.75–0.80]), *p* < 0.001; C4d ρ = 0.86 (95% CI [0.84–0.87]), *p* < 0.001. Download Figure 7-1, EPS file.

Together, our results imply that the subcellular distribution of Gphn C4c shows differences from the other C4 isoforms. On average, fewer Gphn C4c molecules get recruited to P1 predominating clusters. The fluorescence intensities of the corresponding isoforms at each individual synaptic cluster demonstrate this relationship in detail. Spearman correlation analysis revealed monotonic relationships between mEGFP and mScarlet fluorescence intensities. Highest correlation coefficients were obtained for P1 ρ = 0.92 (95% CI [0.91–0.93]) and C4a ρ = 0.92 (95% CI [0.91–0.93]), while the coefficient for C4d was smaller ρ = 0.86 (95% CI [0.84–0.87]) and the smallest for C4c ρ = 0.78 (95% CI [0.75–0.80]; Extended Data Fig. 7-1*b*). Overall, these results suggest that while all C4 isoforms can colocalize with synaptic P1 clusters, C4c molecules were the least abundant at these sites.

### Functional differences in inhibitory signaling between Gphn P1 and Gphn C4c

Our previous experiments suggested that Gphn P1 and Gphn C4c localized differently in cultured hippocampal glutamatergic neurons. We postulated that these gephyrin isoforms might differentially associate with distinct types of GABAergic synapses. GABA_A_R α subunits have been shown to localize to distinct sites. For example, GABA_A_R α1 predominates at dendritic and somatic synapses, while GABA_A_R α2 is found at the axon initial segment (Nusser et al., 1996; Nyíri et al., 2001). However, when we analyzed GABA_A_R α1 cluster size and intensity at Gphn P1 and Gphn C4c clusters, no differences were detected (Extended Data Fig. 8-1*a–c*). Similarly, no differences were observed for GABA_A_R α2 cluster size and intensity at either Gphn isoform (Extended Data Fig. 8-1*d*,*e*).

**Figure 8.**
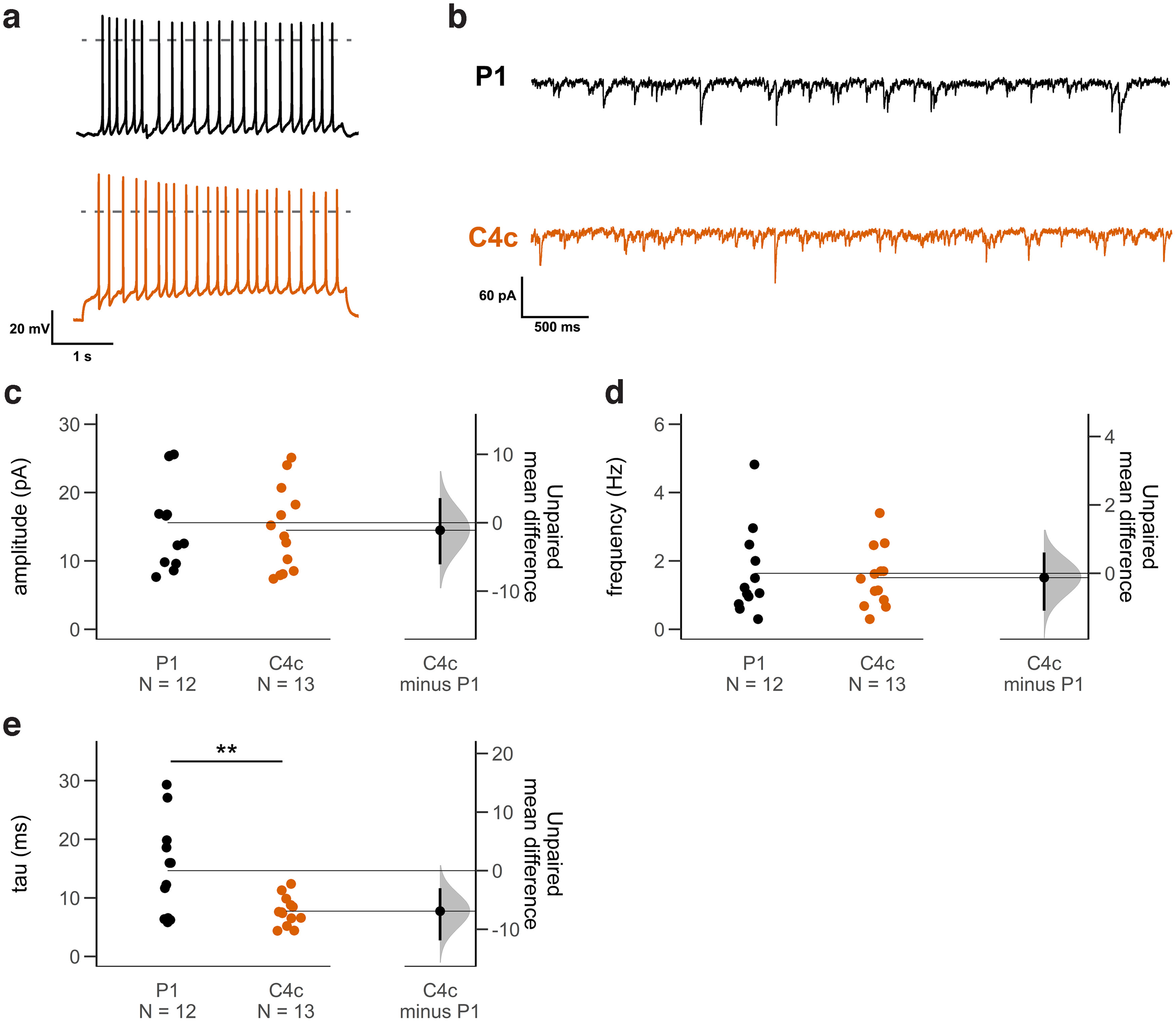
Analysis of miniature IPSCs (mIPSCs) in cultured hippocampal neurons expressing either mScarlet-tagged Gphn P1 or C4c. P1 (black), C4c (orange). ***a***, Current-clamp recordings showing the firing activity of cultured neurons during a 2 s depolarizing current pulse. The dashed lines indicate 0 mV. ***b***, Example voltage clamp recordings of mIPSCs from either splice variant. ***c–e***, Estimation plots for mIPSC amplitude, frequency, and decay of P1 and C4c splice variants. ***c***, There was no significant difference in mIPSC amplitudes between P1 and C4c splice variants [P1: 15.6 ± 1.9 pA, C4c: 14.5 ± 1.7 pA; *N*_P1_ = 12, *N*_C4c_ = 13; unpaired mean difference (ΔC4c-P1): −1.1 pA; 95% CI [−6.1–3.7]; *p*_P1/C4c_ = 0.68 (two-sided permutation test)]. ***d***, There was no significant difference in mIPSC frequencies [P1: 1.6 ± 0.4 Hz, C4c: 1.5 ± 0.2 Hz; *N*_P1_ = 12, *N*_C4c_ = 13; unpaired mean difference (ΔC4c-P1): −0.1 Hz; 95% CI [−1.1–0.6]; *p*_P1/C4c_ = 0.78 (two-sided permutation test)]. ***e***, The decay time constants of the mIPSCs were shorter in C4c compared with P1 [P1: 14.6 ± 2.3 ms, C4c: 7.7 ± 0.7 ms; *N*_P1_ = 12, *N*_C4c_ = 13; unpaired mean difference (ΔC4c-P1): −6.9 ms; 95% CI [−12.1–−3.0]; *p*_P1/C4c_ = 0.0056 (two-sided permutation test)]; **p < 0.01. Each data point represents the mean of a 50 s recording interval of an individual neuron. The filled curves indicate the resampled Δ distribution (5000 bootstrap samples) derived from the observed data. The Δ is indicated by the black circle, which is horizontally aligned with the mean of the test group. The 95% confidence interval of the mean difference is illustrated by the black vertical line. All recordings were performed at 12–15 DIV. During the voltage clamp recordings, glutamatergic input was blocked by CNQX and DL-AP5. Na^+^-mediated action potentials were blocked by TTX. Holding potential was −50 mV. See Extended Data Figure 8-1.

10.1523/ENEURO.0102-22.2022.f8-1 Extended Data Figure 8-1 Expression and analysis of mScarlet-tagged Gphn P1 and C4c isoforms in CamKIIα-expressing murine hippocampal neurons stained for GABA_A_Rα1 and GABA_A_Rα2. ***a***, Hippocampal cultures were transfected after 8 DIV with moxBFP-IRES-Flpo under the control of the CamKIIα promoter. After 9 DIV, cultures were transduced with adeno-associated virus (AAV) 2/1 carrying Flp dependent mScarlet-tagged Gphn P1 or C4c variants as transgene. Cells were immunostained for GABA_A_Rα1 and GABA_A_Rα2 after 15 DIV. Representative confocal images (with adaptive image reconstruction) of neurons expressing moxBFP-IRES-Flpo and mScarlet-tagged Gphn. Scale bars: 25 μm and 2.5 μm in insets. ***b–e***, Estimation plots of GABA_A_Rα1 and GABA_A_Rα2 cluster sizes and fluorescence intensities at P1 and C4c clusters. ***b***, GABA_A_Rα1 cluster sizes (average per cell) are not different at P1 and C4c clusters; unpaired two-samples Wilcoxon test, W = 128, *p* = 0.539. ***c***, GABA_A_Rα1 cluster intensities (average per cell) are not different at P1 and C4c clusters; unpaired two-samples Wilcoxon test, W = 105, *p* = 0.775. ***d***, GABA_A_Rα2 cluster sizes (average per cell) are not different at P1 and C4c clusters; unpaired two-sample *t* test *t*_(28)_ = 1.296, *p* = 0.206. ***e***, GABA_A_Rα2 cluster intensities (average per cell) are not different at P1 and C4c clusters; unpaired two-samples Wilcoxon test, W = 102, *p* = 0.683. Each data point represents the mean for an individual neuron. The filled curves indicate the resampled Δ distribution (5.000 bootstrap samples) derived from the observed data. The Δ is indicated by the black circle, which is horizontally aligned with the mean of the test group. The 95% confidence interval of the mean difference is illustrated by the black vertical line. Download Figure 8-1, TIF file.

Since cluster size and cluster intensities of synaptic GABA_A_Rɣ2 clusters were increased at exogenous Gphn clusters (Extended Data Fig. 4-1*c*,*d*), we next tested whether inhibitory currents were affected. Current-clamp recordings showed that the cultured neurons could generate overshooting Na^+^-mediated action potentials, indicating their healthy physiological state (Fig. 8*a*). When action potentials (by TTX) and glutamatergic receptors (by DL-AP5 and CNQX) were blocked, we observed spontaneous miniature IPSCs (mIPSCs; Fig. 8*b*). A quantitative analysis revealed that neither mIPSC frequency nor mIPSC amplitude were different between Gphn P1 and Gphn C4c expressing cells (Fig. 8*c*,*d*). These results are in agreement with our immunocytochemistry staining, which showed no difference in GABA_A_Rɣ2 clustering and no change in Gphn cluster density between P1 and C4c (Fig. 4*a*,*b*; Extended Data Fig. 3-1*b*). Interestingly, mIPSCs displayed different kinetics, as revealed by faster decay rates in Gphn C4c versus Gphn P1-expressing cells (Fig. 8*e*).

In summary, while Gphn P1 and C4c did not differentially associate with GABA_A_R α1 or GABAR α2 clusters, functional experiments revealed that inhibitory currents decayed faster in the presence of Gphn C4c compared with Gphn P1. These results could be because of the scaffolding of different types of GABA_A_Rs, the recruitment of additional factors that influence GABA_A_R kinetics, or a direct effect of gephyrin clustering on the receptor properties.

## Discussion

Alternative splicing contributes to transcriptome and proteome diversity (Lopez Soto et al., 2019). Since the discovery of Gphn, alternatively spliced transcripts have been reported (Prior et al., 1992; Kirsch et al., 1993; Heck et al., 1997; Ramming et al., 2000; David-Watine, 2001; Rees et al., 2003; Dos Reis et al., 2022). However, for the different neuronal isoforms, functional differences have not been studied in detail. In the SPLICECODE database, Gphn P1 is the most abundant isoform (∼62% of all transcripts) in major glutamatergic and GABAergic neurons in hippocampus and cortex (Furlanis et al., 2019). This suggests that Gphn’s major function as an inhibitory synaptic scaffolding protein can be fulfilled by P1 and cassette exon splicing generates isoforms with likely modulated properties.

Roughly every 10th transcript (9%) contains the cassette G2 (SPLICECODE database), which alters the protein’s trimerization behavior and molybdenum cofactor biosynthesis (Meier et al., 2000; Bedet et al., 2006; Smolinsky et al., 2008). This can be explained by the fact that G2 cassette insertion disrupts ɑ-helix four of the Gphn G-domain (Prior et al., 1992; Schwarz et al., 2001; Sola et al., 2001). In neurons, Gphn G2 can act in a dominant-negative fashion on GlyR clustering, possibly because of its reduced ability to trimerize (Bedet et al., 2006). Therefore, it is conceivable that G2 cassette exon splicing regulates Gphn scaffold remodelling and could contribute to the formation of structurally dynamic postsynaptic clusters.

The presence of ∼1% C3-containing transcripts (SPLICECODE database) could be because of imperfect NOVA splicing (Ule et al., 2003; Licatalosi et al., 2008). In addition, previous reports have associated Gphn C3 with non-neuronal expression and molybdenum cofactor biosynthesis (Ramming et al., 2000; Rees et al., 2003; Paarmann et al., 2006; Smolinsky et al., 2008; Nawrotzki et al., 2012). In fact, in the SPLICECODE database, no evidence for C3 expression in CamKII-expressing neurons was detected and transcript levels were low in the other neuronal cell types. Furthermore, C3 was not expressed in glia-free cortical primary neurons, while abundant in neuron-free glia cultures (Smolinsky et al., 2008).

The remaining 28% of transcripts differ in their C4 cassettes, with the majority of transcripts containing C4a (20%) and minor amounts having C4c (3%) or C4d (5%) incorporated (SPLICECODE database). This implies that the majority of neuronal alternatively spliced gephyrin transcripts are generated through differential incorporation of C4 cassettes. These insertions might provide additional protein-protein binding sites, change binding affinities, or alter the structure, thereby modifying the function of the protein isoforms.

The C4a cassette translates into 19 residues (Heck et al., 1997). In a recent study, where exogenous gephyrin was already introduced at DIV1 into cultured hippocampal neurons, increased cluster sizes were observed for a C4a-containing construct compared with P1 (Dos Reis et al., 2022). In contrast, our analysis of synaptic clusters did not reveal such differences between the two isoforms. A major difference between the two experimental approaches is the time point of exogenous Gphn expression. Based on our analysis of endogenous C4 transcript expression, we introduced exogenous Gphn at a time when endogenous expression had already plateaued. Additionally, our transcript analysis suggested that C4a-containing transcripts are expressed later than C4-lacking ones. Therefore, it is possible that cluster formation is altered if the sequence of expression is reversed (Dos Reis et al., 2022).

Three different residues within the 14 amino acid long C4c cassette can be phosphorylated; in a bioinformatic prediction, multiple kinases such as PKA, PKC, CamKII, GSK3, RSK, and cdc2 have been identified as potential candidates (Herweg and Schwarz, 2012). The introduction of additional phosphorylation sites into gephyrin’s C-domain could add an extra regulatory layer. Phosphorylation at various sites in the central domain have been associated with structural remodeling of GABAergic synapses (Tyagarajan et al., 2011, 2013; Flores et al., 2015). Therefore, it could be postulated that phosphorylation of a less abundant isoform, such as C4c, could affect GABAergic synapse plasticity in a very distinct context or at a subset of specific synapses. Recombinantly produced proteins P1 and C4c showed similar oligomerization behavior and binding affinities for the GlyR β-loop (Herweg and Schwarz, 2012). Our clustering analysis showed that the subcellular localization and recruitment to clusters with the predominating isoform P1 was strongly reduced for C4c. The localization of synaptic clusters indicates that C4c may preferentially fulfill functions in more distal dendritic regions. This observation is in agreement with the reduced intensities at P1 clusters and implies that P1 and C4c may show differential association with components of the transport machinery. Additionally, faster mIPSC decay rates suggested that GABA_A_R function is differently affected by C4c versus P1 scaffolding.

Similarities between the 21 residue C4d sequence and the α-helical dimerization domain 1B of the human keratin 8 were proposed as an additional binding site for protein-protein interactions (Ramming et al., 2000; Rees et al., 2003). In all our analyses, no difference between C4d and P1 clustering was detected. In a recent study, when C4d-containing exogenous gephyrin was already introduced at DIV1, increased cluster size, decreased cluster density, and increased association with GAD-65 were observed (Dos Reis et al., 2022). Similar to the findings for C4a, this suggests that cluster formation can be affected, when the C4d isoform is expressed before the onset of synaptogenesis (Vogel and Marcotte, 2012; Smith et al., 2013; Liu et al., 2016; Aebersold et al., 2018).

Limitations of our study are the exogenous Gphn expression and the use of dissociated cultured hippocampal neurons. While artificial Gphn aggregation, which is well-known in the field, was reduced with our AAV expression system, we still introduced additional transcript copies. These could promote the association of exogenously expressed Gphn molecules to any preexisting synaptic cluster rather than to a specifically-targeted synapse, simply because of increased protein levels. While our P1-targeting approach underline this view, the results demonstrate that C4c-containing variants were less abundant at P1 clusters, which is in line with the preferred distal dendritic localization of this variant.

Regarding a second limitation, dissociated neuron cultures may not retain their full set of naturally occurring synaptic partners. For instance, different types of interneurons can preferentially form synapses in specific regions of the postsynaptic neuron; SST-expressing neurons predominantly from synapses on dendrites, while parvalbumin-expressing neurons form synapses in the perisomatic region (Tremblay et al., 2016; Contreras et al., 2019). While some aspects of subcellular postsynaptic protein localization can occur in a cell autonomous fashion, i.e., in dissociated cultures, others may only be achieved when the correct anatomic input-specificity is preserved, i.e., *in vivo*.

Importantly, in our experimental paradigm, exogenous Gphn expression started roughly after DIV11, which is later than the onset of synaptogenesis (Deng et al., 2007; Grabrucker et al., 2009). Therefore, our observations relate to the scaffolding of Gphn isoforms in neurons with preexisting inhibitory synapses and no conclusions can be made about the impact of C4 alternative splicing on synaptogenesis. To advance our knowledge about the functional impact of C4 splicing, analyses of the scaffolding behavior of endogenously expressed gephyrin C4 isoform are needed. Here, a genetic deletion of individual C4 cassettes is needed, which is similar to an approach that was recently performed for an alternatively spliced exon of Neurexin 3 (Hauser et al., 2022). In addition, the use of neurons with a complete deletion of endogenous Gphn and subsequent expression of individual Gphn variants will further isolate functions of each Gphn splice variants.

Overall, cassette exon splicing of the C4 cluster occurred in GABAergic and glutamatergic neurons of the hippocampus and cortex (SPLICECODE dataset) and other studies have suggested that this is predominantly a neuron-specific process (Ramming et al., 2000; Rees et al., 2003; Paarmann et al., 2006; Smolinsky et al., 2008). Exogenously expressed C4 isoforms in glutamatergic dissociated neurons demonstrated, for the most part, redundant scaffolding behavior, while localization and functional differences for C4c-containing isoforms suggest that C4 cassette splicing can indeed contribute to inhibitory synapse heterogeneity.

## Synthesis

Reviewing Editor: Alexey Semyanov, Shemyakin-Ovchinnikov Institute of Bioorganic Chemistry Decisions are customarily a result of the Reviewing Editor and the peer reviewers coming together and discussing their recommendations until a consensus is reached. When revisions are invited, a fact-based synthesis statement explaining their decision and outlining what is needed to prepare a revision will be listed below. The following reviewer(s) agreed to reveal their identity: Rochelle Hines.

The manuscript addresses a timely question on how alternative splicing of gephyrin contributes to GABAergic synapse diversity. The key observation is that including the C4c cassette affects the distribution of GABAA receptors along the dendrite is interesting to neurophysiologists. However, a single research method represents the major limitation of the study.

The reviewers pointed out that the functional relevance of the findings is missing. Addressing several unanswered questions would also strengthen the manuscript. For example, the subcellular distribution of GABAA receptors depends on their subunit composition. It is important to investigate whether the insertion of the C4 cassette affects the subunit composition of GABAA receptors. A linked question is the physiological consequences of receptor redistribution and possible change in their properties. This could affect spontaneous IPSCs. A reviewer suggested looking at mIPSC. In parallel, tonic current mediated by extrasynaptic GABAA receptor current can be measured.

Attention is also needed in the statistics section.

Reviewers’ comments

Reviewer 1

In the present manuscript, the authors address the question whether alternative splicing of the GABAergic postsynaptic scaffolding protein gephyrin regulates its localization to GABAergic synapses and hence may contribute to GABAergic synapse diversity. In particular, the authors focus on one set of alternatively spliced cassettes, the C4 cassettes. To this end, the authors first investigate the expression pattern of isoforms containing the C4a, C4c and C4d cassettes using the SPLICECODE database as well as PCR analysis in hippocampal cultures. They then overexpressed gephyrin constructs containing the C4a, C4c or C4d cassettes in hippocampal cultures and compared them to a gephyrin construct containing no C4 cassette. They showed that none of the splice cassettes have a significant impact on the number, size, intensity or GABAARg2 content of synaptic gephyrin clusters, although the distribution along the dendrite was changed by inclusion of the C4c cassette. Moreover, the gephyrin construct containing the C4c cassette showed reduced colocalization with the construct containing no C4 cassette, indicating differential localization mechanisms. Together, the authors show that inclusion of the C4c cassette, but not of the C4a or C4d cassettes, regulates the localization of gephyrin at specific GABAergic synapses.

Overall, this manuscript addresses a very timely question, i.e. the molecular mechanisms that underlie the structural and functional diversity of GABAergic synapses, and the results are potentially interesting to a widespread community of synapse biologists. The experiments appear carefully and thoroughly conducted, and the manuscript is very well written. However, the data are rather limited in scope, essentially consisting of only two experiments using one methodology, i.e. virally mediated expression of gephyrin constructs in hippocampal cultures, followed by immunohistochemical analysis of gephyrin localization. This leaves quite a few unanswered questions, some of which the authors address in their discussion. Addressing these questions would greatly strengthen the manuscript.

The most obvious and pressing question regards the effects of the C4 cassette on GABAergic synapse composition and synaptic transmission. While the authors do show initially that the colocalization of gephyrin with the GABAAR subunit gamma2 is not affected by inclusion of the C4 cassettes, they make no further efforts to investigate the consequences of the C4 cassettes for GABAAR recruitment. In particular, the following questions should be addressed (ideally in a background in which endogenous gephyrin has been knocked down):

(1) Is any shift in the distribution of the GABAAR gamma 2 subunit observed, analogous to the shift toward more distal localization of the C4c-containing gephyrin construct, or is there a difference in the size and intensity of GABAAR gamma 2 subunit clusters that colocalize with the different gephyrin clusters? Some of these issues are addressed in a supplemental figure, which should be moved to the main text and expanded.

(2) Is there a change in the subunit composition of GABAARs, i.e. are there alterations in distribution, cluster size or intensity of any of the alpha or beta subunits? This would be critical for knowing whether a shirt in the functional properties of GABAARs may be induced by the C4 cassette.

(3) Are there effects on GABAergic synaptic transmission, specifically on mIPSCs? It is conceivable that the C4 cassettes have consequences for GABAAR function that are not solely related to localization at the synapse.

These data should be added to the manuscript, since without these answers, it is challenging to assess the relevance of the findings described here.

A further limitation (which the authors already address in the discussion) is that the specificity for different GABAergic synapse subtypes is not investigated. The GABAergic system is highly diverse, and the complex endogenous connectivity cannot be fully mimicked in dissociated cultures. Nevertheless, one aspect that could be investigated in the cultures is to assess whether synapses formed by different GABAergic neuron subtypes (e.g. parvalbumin, somatostatin, VIP) show differential localization of the different gephyrin isoforms. This can be done by using cell type specific Cre driver lines to tag the individual neuron subtypes. Or in the absence of these Cre driver lines, recent efforts have identified AAV-compatible promoter elements that restrict expression at least to PV-positive neurons (Vormstein-Schneider et al. 2020, Nat Neurosci 23:1629). In combination with a Cre-dependent presynaptic marker, these could be used to investigate input-specific isoform distribution at least for PV-positive inputs.

Reviewer 2

The submitted manuscript describes the development and testing of an automated ImageJ script for the analysis of synaptic protein splice variant localization in cultured neurons. The work builds off of the available SPLICECODE DATABASE information for the inhibitory synaptic scaffold gephyrin, and goes on to analyze the subcellular localization of C4 cassette containing variants. This work is of interest due to the known heterogeneity of specialized inhibitory synapse subtypes, yet the seemingly ubiquitous inclusion of gephyrin at these sites. One possible contribution to inhibitory synapse heterogeneity could be the specific gephyrin isoform localized to these sites. Understanding these mechanisms could lead to novel methods of specifically regulating inhibitory synapse subtypes.

Strengths of the work

The work is well executed, and employs appropriate methods to assess the localization of C4 cassette containing isoforms of gephyrin compared to the abundant P1 isoform that lacks additional cassettes.

The work develops a new script available to use in the freely available and widely used ImageJ platform. This will enable other researchers to apply this tool to their studies.

Limitations of the work:

While the work provides insight on possible enriched localization of gephyrin C4 splice variants, these are overstated, and implications for function or examination of mechanism remain unexplored.

Suggestions for revision:

For the analysis performed in figure 3f it would also be interesting to examine the within C4 variant localization. For example, it appears as though C4a is significantly more enriched in perisomatic sites, compared to the proximal dendrites (and maybe higher again at distal sites)? Similarly, it appears that C4c and C4d are both preferentially enriched distally as compared to perisomatic and proximal sites. This seems to be an important set of comparisons to examine.

It is not apparent to me that the slight decrease in slope suggests “exclusion” of C4c from P1 positive clusters. If the relationship were exclusive one would anticipate that mEGFP cluster intensity would be highest when mScarlet cluster intensity was lowest (inverse relationship). What is observed is only a slight shift in slope. So, this interpretation of what the slope change implies needs to be revised accordingly.

Inclusion of phosphorylation sites is mentioned briefly as a possible mechanism, but this is only very lightly explored in the discussion. Related, the whole paragraph on PKC phosphorylation of the C4a cassette is a little confusing as worded (p 18, lines 391-396). It states both that “no differences” were observed, and also that the cluster by cluster analysis revealed that the “slope was changed”. These two statements are in conflict with one another. Interpretation of what the change (maybe increase) in slope means would also be useful here (as perhaps already implied from above statement).

Also, for the C4c cassette discussion of phosphorylation sites is brought up but only very superficially discussed. Making a more concrete connection between possible phosphorylation state and transport is needed to strengthen this discussion. Also, what implications might phosphorylation have on the function of gephyrin at the distal inhibitory synaptic sites?

The discussion raises a few of the limitations, but this aspect would also be strengthened by identifying what is needed next to advance the literature.

Additional information is needed in the methods:

-the paper mentions that 60 cells were analyzed, but from how many independent cultures / replicates?

-additional information on imaging (numerical aperture, actual resolution of acquired images beyond microscope capacity...)

-additional general information about statistics is needed (as noted above)

Minor suggestions:

I like and appreciate the graphics / icons in the figures - they are appealing, but in some cases, they are used in exchange for adequate information about what the figure is showing. Although the captions are helpful, it is desirable to have a reader be able to interpret the data independent of the text and captions.

Some of the text on the axes or on the icons are extremely small. The figures will also likely need to be made even smaller (or adjusted to different aspect ratios) as it is unlikely that whole pages of the pdf will be devoted to each figure. Please increase the size of the text.

p 4 lines 84-86 - the articulation of the hypothesis is awkward / syntactical error

p 30 line 722 - the caption states hippocampus but extended Figure 1-1 shows cortex (stated on figure and earlier in caption).

As written, those unfamiliar with R would likely find the statistics section useless - is not prepared with sufficient information to determine if the statistics were properly applied (specific tests used, prerequisites / assumptions, significance levels, post hoc tests). Meanwhile there is a lot of stats section info in the figure captions. My recommendation would be to make the statistics section more readable and informative with general information about procedures, and put the specific details of each analysis relevant to each graph in the captions. The statistics section is also a good place to describe the data transformations used to generate the correlation plots rather than in the results section.

Extended data figure 1-1 is useful and appropriate (shows cortex for comparison, which is complementary to the hippocampal focus of the paper but not necessary within the figure). For the others (3-1, 3-2, 5-1) it is not immediately clear why these are extended data... other “not statistically significant” plots are shown in the main figures, why were these chosen to be in extended data?

## Author Response

Synthesis Statement for Author (Required):

The manuscript addresses a timely question on how alternative splicing of gephyrin contributes to GABAergic synapse diversity. The key observation is that including the C4c cassette affects the distribution of GABAA receptors along the dendrite is interesting to neurophysiologists. However, a single research method represents the major limitation of the study.

The reviewers pointed out that the functional relevance of the findings is missing.

Addressing several unanswered questions would also strengthen the manuscript.

For example, the subcellular distribution of GABAA receptors depends on their subunit composition. It is important to investigate whether the insertion of the C4 cassette affects the subunit composition of GABAA receptors. A linked question is the physiological consequences of receptor redistribution and possible change in their properties. This could affect spontaneous IPSCs. A reviewer suggested looking at mIPSC. In parallel, tonic current mediated by extrasynaptic GABAA receptor current can be measured.

Attention is also needed in the statistics section.

ANSWER:

We thank the reviewers for their constructive criticism regarding our manuscript entitled “Automated image analysis reveals different localization of synaptic gephyrin

C4 splice variants”. We have addressed all points raised (see below) and highlighted changes in the manuscript text. Additionally, we updated specific sections of the manuscript due to a recent publication on the alternative slicing of Gephyrin (Dos Reis, et al. 2022). Furthermore, we included estimation statistics and replaced figures with Gardner-Altman plots and Cummings estimation plots in accordance with eNeuro’s initiative to present results with estimation statistics.

Reviewers’ comments

Reviewer 1

In the present manuscript, the authors address the question whether alternative splicing of the GABAergic postsynaptic scaffolding protein gephyrin regulates its localization to GABAergic synapses and hence may contribute to GABAergic synapse diversity. In particular, the authors focus on one set of alternatively spliced cassettes, the C4 cassettes. To this end, the authors first investigate the expression pattern of isoforms containing the C4a, C4c and C4d cassettes using the SPLICECODE database as well as PCR analysis in hippocampal cultures. They then overexpressed gephyrin constructs containing the C4a, C4c or C4d cassettes in hippocampal cultures and compared them to a gephyrin construct containing no C4 cassette. They showed that none of the splice cassettes have a significant impact on the number, size, intensity or GABAARg2 content of synaptic gephyrin clusters, although the distribution along the dendrite was changed by inclusion of the C4c cassette. Moreover, the gephyrin construct containing the C4c cassette showed reduced colocalization with the construct containing no C4 cassette, indicating differential localization mechanisms. Together, the authors show that inclusion of the C4c cassette, but not of the C4a or C4d cassettes, regulates the localization of 2 gephyrin at specific GABAergic synapses.

Overall, this manuscript addresses a very timely question, i.e. the molecular mechanisms that underlie the structural and functional diversity of GABAergic synapses, and the results are potentially interesting to a widespread community of synapse biologists. The experiments appear carefully and thoroughly conducted, and the manuscript is very well written. However, the data are rather limited in scope, essentially consisting of only two experiments using one methodology, i.e. virally mediated expression of gephyrin constructs in hippocampal cultures, followed by immunohistochemical analysis of gephyrin localization. This leaves quite a few unanswered questions, some of which the authors address in their discussion.

Addressing these questions would greatly strengthen the manuscript.

ANSWER:

We thank the Reviewer#1 for the feedback. We are pleased that R#1 feels that our study addresses a very timely question. We are confident that we have clearly improved the revised version of the manuscript based on her/his suggestions and addressed all her/his concerns by providing the additional explanations as well as additional experimental data in our point-to-point reply as follows.

The most obvious and pressing question regards the effects of the C4 cassette on GABAergic synapse composition and synaptic transmission. While the authors do show initially that the colocalization of gephyrin with the GABAAR subunit gamma2 is not affected by inclusion of the C4 cassettes, they make no further efforts to investigate the consequences of the C4 cassettes for GABAAR recruitment. In particular, the following questions should be addressed (ideally in a background in which endogenous gephyrin has been knocked down): (1) Is any shift in the distribution of the GABAAR gamma 2 subunit observed, analogous to the shift toward more distal localization of the C4c-containing gephyrin construct, or is there a difference in the size and intensity of GABAAR gamma 2 subunit clusters that colocalize with the different gephyrin clusters? Some of these issues are addressed in a supplemental figure, which should be moved to the main text and expanded.

ANSWER:

To answer the reviewer’s question, we provide additional analyses of our data and summarized our findings in the new main Fig. 4, Extended Data Fig. 4-1, and, Extended Data Fig. 5-1a. We find that GABAAR ɣ2 clusters are larger at Gphn C4a vs C4d and as expected by the reviewer, the localization shift of GABAAR ɣ2 clusters is analogous to the shift that we observed for Gphn clusters.

"Next, we analyzed whether the expression of Gphn C4 isoforms affected GABAAR clustering. When we selected synaptic GABAARɣ2 clusters (vGAT-positive), larger clusters were observed at Gphn C4a compared with Gphn C4d, while all other comparisons did not reveal differences (Fig. 4a; Extended Data Fig. 4a). Additionally, 3 no differences in GABAARɣ2 cluster intensities at the different C4 isoform clusters were observed (Fig. 4b; Extended Data Fig. 4-1b). Importantly, the cluster size and cluster intensities of synaptic GABAARɣ2 clusters was increased at all exogenously expressed C4 isoforms compared with endogenous clusters (Extended Data Fig. 4- 1c, d).” ...

In agreement with this, larger distances were detected for GABAARɣ2 clusters localized at isoform C4c compared with P1 and C4a, while the difference between C4c and C4d was not statistically significant (Extended Data Fig. 5-1a). (2) Is there a change in the subunit composition of GABAARs, i.e. are there alterations in distribution, cluster size or intensity of any of the alpha or beta subunits? This would be critical for knowing whether a shirt in the functional properties of GABAARs may be induced by the C4 cassette.

ANSWER:

We thank the reviewer for this comment. Since especially GABAARα1 and GABAARα2 have been described to localize to distinct sites, we tested, whether Gphn showed any preference for either of these clusters. Since our previous data revealed mainly localization differences between Gphn P1 and Gphn C4c, we focussed our experiments on these two isoforms. The analysis did not reveal any difference between GABAARα1 or GABAARα2 cluster size or intensity at either of the two isoforms. Our additional data can be found in Extended Fig. 8-1.

"Our previous experiments suggested that Gphn P1 and Gphn C4c localized differently in cultured hippocampal glutamatergic neurons. We postulated that these gephyrin isoforms might differentially associate with distinct types of GABAergic synapses. GABAAR α subunits have been shown to localize to distinct sites. For example, GABAAR α1 predominates at dendritic and somatic synapses, while GABAAR α2 is found at the axon initial segment (Nusser et al., 1996; Nyiri et al., 2001). However, when we analyzed GABAAR α1 cluster size and intensity at Gphn P1 and Gphn C4c clusters, no differences were detected (Extended Data Fig. 8-1a- 4 c). Similarly, no differences were observed for GABAAR α2 cluster size and intensity at either Gphn isoform (Extended Data Fig. 8-1d,e).”

(3) Are there effects on GABAergic synaptic transmission, specifically on mIPSCs? It is conceivable that the C4 cassettes have consequences for GABAAR function that are not solely related to localization at the synapse.

These data should be added to the manuscript, since without these answers, it is challenging to assess the relevance of the findings described here.

ANSWER:

The reviewer’s question is indeed very important to our line of research. To test whether C4 splicing results in functional differences, we now provide new electrophysiology experiments to measure mIPSCs. To focus our experiments, we tested differences between Gphn P1 and C4c (P2). While no differences between mIPSC amplitude and frequency were observed (in line with our immunocytochemistry findings), faster deactivation (as indicated by smaller deactivation constant tau) for Gphn C4c was observed. These findings suggest that the two isoforms have a functional impact on GABAARs. The additional experiments can be found in Fig. 8.

"Since cluster size and cluster intensities of synaptic GABAARɣ2 clusters were increased at exogenous Gphn clusters (Extended Data Fig. 4-1c, d), we next tested whether inhibitory currents were affected. Current clamp recordings showed that the cultured neurons could generate overshooting Na+ -mediated action potentials, indicating their “healthy physiological state (Fig. 8a). When action potentials (by TTX) and glutamatergic receptors (by DL-AP5 and CNQX) were blocked, we observed spontaneous miniature inhibitory postsynaptic currents (mIPSCs) (Fig. 8b). A quantitative analysis revealed that neither mIPSC frequency nor mIPSC amplitude were different between Gphn P1 and Gphn C4c expressing cells (Fig. 8c,d). These results are in agreement with our immunocytochemistry staining, which showed no difference in GABAARɣ2 clustering and no change in Gphn cluster density between P1 and C4c (Fig. 4a,b, Extended Data Fig. 3-1b). Interestingly, mIPSCs displayed 5 different kinetics, as revealed by faster decay rates in Gphn C4c vs Gphn P1 expressing cells (Fig. 8e).

In summary, while Gphn P1 and C4c did not differentially associate with GABAAR α1 or GABAR α2 clusters, functional experiments revealed that inhibitory currents decayed faster in the presence of Gphn C4c compared with Gphn P1. These results could be due to the scaffolding of different types of GABAARs, the recruitment of additional factors that influence GABAAR kinetics, or a direct effect of gephyrin clustering on the receptor properties.”

A further limitation (which the authors already address in the discussion) is that the specificity for different GABAergic synapse subtypes is not investigated. The GABAergic system is highly diverse, and the complex endogenous connectivity cannot be fully mimicked in dissociated cultures. Nevertheless, one aspect that could be investigated in the cultures is to assess whether synapses formed by different GABAergic neuron subtypes (e.g. parvalbumin, somatostatin, VIP) show differential localization of the different gephyrin isoforms. This can be done by using cell type specific Cre driver lines to tag the individual neuron subtypes. Or in the absence of these Cre driver lines, recent efforts have identified AAV-compatible promoter elements that restrict expression at least to PV-positive neurons (Vormstein-Schneider et al. 2020, Nat Neurosci 23:1629). In combination with a Cre-dependent presynaptic marker, these could be used to investigate input-specific isoform distribution at least for PV-positive inputs.

ANSWER:

We thank the reviewer for this suggestion. We agree that it would be interesting to know, whether Gphn isoforms preferably associate to specific GABAergic synapses formed by different presynaptic neurons. We will focus our future research efforts on this question; however, we believe that our current model system (hippocampal neurons DIV15) may not be ideal to satisfactorily answer this question. As stated in the publication referred to by the reviewer, PV is expressed fairly late in postnatal development and although the author’s AAV-compatible promoter element improved early labeling of PV neurons in vivo, reporter expression was predominantly found in the cortex (Vormstein-Schneider et al., 2020).

For this reason, our relatively immature hippocampal neurons may not be the best model system to answer this interesting question. At the current state and with the tools at our disposal, the search for an association of Gphn isoforms with specific presynaptic neurons is beyond the scope of this manuscript. Reviewer 2 6

The submitted manuscript describes the development and testing of an automated ImageJ script for the analysis of synaptic protein splice variant localization in cultured neurons. The work builds off of the available SPLICECODE DATABASE information for the inhibitory synaptic scaffold gephyrin, and goes on to analyze the subcellular localization of C4 cassette containing variants. This work is of interest due to the known heterogeneity of specialized inhibitory synapse subtypes, yet the seemingly ubiquitous inclusion of gephyrin at these sites. One possible contribution to inhibitory synapse heterogeneity could be the specific gephyrin isoform localized to these sites. Understanding these mechanisms could lead to novel methods of specifically regulating inhibitory synapse subtypes.

Strengths of the work

The work is well executed, and employs appropriate methods to assess the localization of C4 cassette containing isoforms of gephyrin compared to the abundant P1 isoform that lacks additional cassettes. The work develops a new script available to use in the freely available and widely used ImageJ platform. This will enable other researchers to apply this tool to their studies.

Limitations of the work:

While the work provides insight on possible enriched localization of gephyrin C4 splice variants, these are overstated, and implications for function or examination of mechanism remain unexplored.

ANSWER:

We thank the Reviewer#2 for the feedback. We are pleased that R#2 feels that our work is well executed and that we used appropriate methods to answer our research questions. We are confident that we have clearly improved the revised version of the manuscript based on the suggestions with additional experiments and modified statements as outlined below.

Suggestions for revision:

For the analysis performed in figure 3f it would also be interesting to examine the within C4 variant localization. For example, it appears as though C4a is significantly more enriched in perisomatic sites, compared to the proximal dendrites (and maybe higher again at distal sites)? Similarly, it appears that C4c and C4d are both preferentially enriched distally as compared to perisomatic and proximal sites. This seems to be an important set of comparisons to examine.

ANSWER:

We thank the reviewer for the suggestion and we find indeed that while P1 clusters showed no preference for the distances, C4a showed a higher fraction at perisomatic and distal sites. Additionally, both C4c and C4d showed a higher fraction at distal sites. We have added the analysis to our manuscript in new Extended Data Fig. 5- 1b. 7

"When the effect was analyzed at each isoform, the fraction of synaptic P1 clusters showed no difference between the distances. Synaptic C4a clusters showed a higher fraction of perisomatic clusters (r < 20 μm) and distally located clusters (r > 40 μm) compared with the intermediate location (20 - 40 μm). Both synaptic C4c and C4d clusters showed a higher fraction of distally located clusters (r > 40 μm) compared with the other locations (Extended Data Fig. 5-1b).”

It is not apparent to me that the slight decrease in slope suggests “exclusion” of C4c from P1 positive clusters. If the relationship were exclusive one would anticipate that mEGFP cluster intensity would be highest when mScarlet cluster intensity was lowest (inverse relationship). What is observed is only a slight shift in slope. So, this interpretation of what the slope change implies needs to be revised accordingly.

ANSWER: We thank the reviewer for this comment. We have removed the word excluded, this sentence is now as follows:

"Overall, these results suggest that while all C4 isoforms can colocalize with synaptic P1 clusters, C4c molecules were the least abundant at these sites.” Inclusion of phosphorylation sites is mentioned briefly as a possible mechanism, but this is only very lightly explored in the discussion. Related, the whole paragraph on PKC phosphorylation of the C4a cassette is a little confusing as worded (p 18, lines 391-396). It states both that “no differences” were observed, and also that the cluster by cluster analysis revealed that the “slope was changed”. These two statements are in conflict with one another. Interpretation of what the change (maybe increase) in slope means would also be useful here (as perhaps already implied from above statement).

ANSWER:

We are grateful for the reviewer’s comment. Since there is no experimental evidence which would suggest that a residue in C4a gets phosphorylated, we have removed this aspect of the discussion. Additionally, we changed the analysis of the cluster by cluster to a non-parametric correlation analysis, which indicates comparable strong monotonic relationships for P1 and C4a. In the discussion we now focus on recent experiments that suggest increased C4a clusters, when the isoform was introduced into cultured neurons already at DIV1 (Dos Reis et al., 2022). We have modified the section as follows: 8

"The C4a cassette translates into 19 residues (Heck et al., 1997). In a recent study, where exogenous gephyrin was already introduced at DIV1 into cultured hippocampal neurons, increased cluster sizes were observed for a C4a-containing construct compared with P1 (Dos Reis et al., 2022). In contrast, our analysis of synaptic clusters did not reveal such differences between the two isoforms. A major difference between the two experimental approaches is the timepoint of exogenous Gphn expression. Based on our analysis of endogenous C4 transcript expression, we introduced exogenous Gphn at a time when endogenous expression had already plateaued. Additionally, our transcript analysis suggested that C4a-containing transcripts are expressed later than C4-lacking ones. Therefore, it is possible that cluster formation is altered if the sequence of expression is reversed (Dos Reis et al., 2022).”

Also, for the C4c cassette discussion of phosphorylation sites is brought up but only very superficially discussed. Making a more concrete connection between possible phosphorylation state and transport is needed to strengthen this discussion. Also, what implications might phosphorylation have on the function of gephyrin at the distal inhibitory synaptic sites?

ANSWER:

We thank the reviewer for bringing this to our attention. We expanded the paragraph on the possible impact of C4c phosphorylation.

"The introduction of additional phosphorylation sites into gephyrin’s C-domain could add an extra regulatory layer. Phosphorylation at various sites in the central domain have been associated with structural remodeling of GABAergic synapses (Flores et al., 2015; Tyagarajan et al., 2013; Tyagarajan et al., 2011). Therefore, it could be postulated that phosphorylation of a less abundant isoform, such as C4c, could affect GABAergic synapse plasticity in a very distinct context or at a subset of specific synapses.” 9

The discussion raises a few of the limitations, but this aspect would also be strengthened by identifying what is needed next to advance the literature.

ANSWER:

We are grateful that the reviewer mentions this to us. We have added a section to the paragraph:

"To advance our knowledge about the functional impact of C4 splicing, analyses of the scaffolding behavior of endogenously expressed gephyrin C4 isoform are needed. Here, a genetic deletion of individual C4 cassettes is needed, which is similar to an approach that was recently performed for an alternatively spliced exon of Neurexin 3 (Hauser et al., 2022). In addition, the use of neurons with a complete deletion of endogenous Gphn and subsequent expression of individual Gphn variants will further isolate functions of each Gphn splice variants.”

Additional information is needed in the methods: -the paper mentions that 60 cells were analyzed, but from how many independent cultures / replicates?

ANSWER:

We thank the reviewer the comment. We have expanded the respective methods sections.

"For imaging experiments, N = 15 cells per condition from three independent cultures were analyzed. For electrophysiology experiments, N = 12 for P1 and N = 13 for C4c cells from five independent cultures were analyzed.” -additional information on imaging (numerical aperture, actual resolution of acquired images beyond microscope capacity...)

ANSWER:

Here, we added more information on the imaging setup. 10

"Image stacks (0.3 μm z-step size, 3,432 x 3,432 (144.77 μm x 144.77 μm)) were acquired on a Leica TCS SP8 LIGHTNING upright confocal microscope with an HC PL APO CS2 63x/1.30 Glyc objective, equipped with hybrid detectors (Leica HyD) and the following diode lasers: 405 nm, 488 nm, 552 nm, and 638 nm. LIGHTNING adaptive deconvolution using ‘Mowiol’ setting were applied. This form of adaptive image reconstruction is capable of theoretical resolutions down to120 nm (lateral) and 200 nm (axial).” -additional general information about statistics is needed (as noted above)

ANSWER:

We added a general paragraph about the statistical analyses.

"Individual data points, mean, confidence intervals, and standard deviation are displayed in the figures. Estimation statistics and plots were generated with the R package DABESTR (Ho et al., 2019). Data were tested for normality, using violation of the Shapiro-Wilk test at p < 0.01 as the criterion. Data were analyzed for homoscedasticity, using violation of Levene’s test at p < 0.01. Data sets not meeting normality or homoscedasticity assumptions were analyzed using non-parametric tests (as indicated in the figure legends). All other data were analyzed with the indicated parametric tests. For imaging experiments, N = 15 cells per condition from three independent cultures were analyzed. For electrophysiology experiments, N = 12 for P1 and N = 13 for C4c cells from five independent cultures were analyzed.”

Minor suggestions:

I like and appreciate the graphics / icons in the figures - they are appealing, but in some cases, they are used in exchange for adequate information about what the figure is showing. Although the captions are helpful, it is desirable to have a reader be able to interpret the data independent of the text and captions. 11

Some of the text on the axes or on the icons are extremely small. The figures will also likely need to be made even smaller (or adjusted to different aspect ratios) as it is unlikely that whole pages of the pdf will be devoted to each figure. Please increase the size of the text.

ANSWER:

We thank the reviewer for alerting us to that. We have reworked all figures and labels. p 4 lines 84-86 - the articulation of the hypothesis is awkward / syntactical error

ANSWER:

We changed the sentence to:

"We hypothesized that C4 isoforms could form structurally different scaffolds, which could in turn affect receptor clustering and/or synapse localization.” p 30 line 722 - the caption states hippocampus but extended Figure 1-1 shows cortex (stated on figure and earlier in caption).

ANSWER:

We changed the respective caption to cortex.

As written, those unfamiliar with R would likely find the statistics section useless - is not prepared with sufficient information to determine if the statistics were properly applied (specific tests used, prerequisites / assumptions, significance levels, post hoc tests). Meanwhile there is a lot of stats section info in the figure captions. My recommendation would be to make the statistics section more readable and informative with general information about procedures, and put the specific details of each analysis relevant to each graph in the captions. The statistics section is also a good place to describe the data transformations used to generate the correlation plots rather than in the results section.

ANSWER:

We reworked the Statistical analysis and data visualization section and included general information to our statistical analyses.

"Data were tested for normality, using violation of the Shapiro-Wilk test at p < 0.01 as the criterion. Data were analyzed for homoscedasticity, using violation of Levene’s test at p < 0.01. Data sets not meeting normality or homoscedasticity 12 assumptions were analyzed using non-parametric tests (as indicated in the figure legends). All other data were analyzed with the indicated parametric tests.” Extended data figure 1-1 is useful and appropriate (shows cortex for comparison, which is complementary to the hippocampal focus of the paper but not necessary within the figure). For the others (3-1, 3-2, 5-1) it is not immediately clear why these are extended data... other “not statistically significant” plots are shown in the main figures, why were these chosen to be in extended data?

ANSWER:

We have reworked all figures and moved all supportive data to the Extended data and all main findings (irrespective of statistical significance) to the main figures. 13

References:

Dos Reis, R., Kornobis, E., Pereira, A., et al. (2022). Complex regulation of Gephyrin splicing is a determinant of inhibitory postsynaptic diversity. Nat Commun, 13(1), 3507. doi:10.1038/s41467-022-31264-w

Flores, C. E., Nikonenko, I., Mendez, P., et al. (2015). Activity-dependent inhibitory synapse remodeling through gephyrin phosphorylation. Proc Natl Acad Sci U S A, 112(1), E65-72. doi:10.1073/pnas.1411170112

Hauser, D., Behr, K., Konno, K., et al. (2022). Targeted proteoform mapping uncovers specific Neurexin-3 variants required for dendritic inhibition. Neuron, 110(13), 2094-2109 e2010. doi:10.1016/j.neuron.2022.04.017

Heck, S., Enz, R., Richter-Landsberg, C., et al. (1997). Expression and mRNA splicing of glycine receptor subunits and gephyrin during neuronal differentiation of P19 cells in vitro, studied by RT-PCR and immunocytochemistry. Brain Res Dev Brain Res, 98(2), 211-220. doi:10.1016/s0165-3806(96)00181-2

Ho, J., Tumkaya, T., Aryal, S., et al. (2019). Moving beyond P values: data analysis with estimation graphics. Nat Methods, 16(7), 565-566. doi:10.1038/s41592- 019-0470-3

Nusser, Z., Sieghart, W., Benke, D., et al. (1996). Differential synaptic localization of two major gamma-aminobutyric acid type A receptor alpha subunits on hippocampal pyramidal cells. Proc Natl Acad Sci U S A, 93(21), 11939-11944. doi:10.1073/pnas.93.21.11939

Nyiri, G., Freund, T. F., & Somogyi, P. (2001). Input-dependent synaptic targeting of alpha(2)-subunit-containing GABA(A) receptors in synapses of hippocampal pyramidal cells of the rat. Eur J Neurosci, 13(3), 428-442. doi:10.1046/j.1460- 9568.2001.01407.x

Tyagarajan, S. K., Ghosh, H., Yevenes, G. E., et al. (2013). Extracellular signal regulated kinase and glycogen synthase kinase 3beta regulate gephyrin postsynaptic aggregation and GABAergic synaptic function in a calpain dependent mechanism. J Biol Chem, 288(14), 9634-9647. doi:10.1074/jbc.M112.442616

Tyagarajan, S. K., Ghosh, H., Yevenes, G. E., et al. (2011). Regulation of GABAergic synapse formation and plasticity by GSK3beta-dependent phosphorylation of gephyrin. Proc Natl Acad Sci U S A, 108(1), 379-384. doi:10.1073/pnas.1011824108

Vormstein-Schneider, D., Lin, J. D., Pelkey, K. A., et al. (2020). Viral manipulation of functionally distinct interneurons in mice, non-human primates and humans. Nat Neurosci, 23(12), 1629-1636. doi:10.1038/s41593-020-0692-9
