# Supplementary figures and images for: Automated Image Analysis Reveals Different Localization of Synaptic Gephyrin C4 Splice Variants
Source: eNeuro. 2023 Jan 3;10(1):ENEURO.0102-22.2022. doi: 10.1523/ENEURO.0102-22.2022 (PMC9831149; doi:10.1523/ENEURO.0102-22.2022)

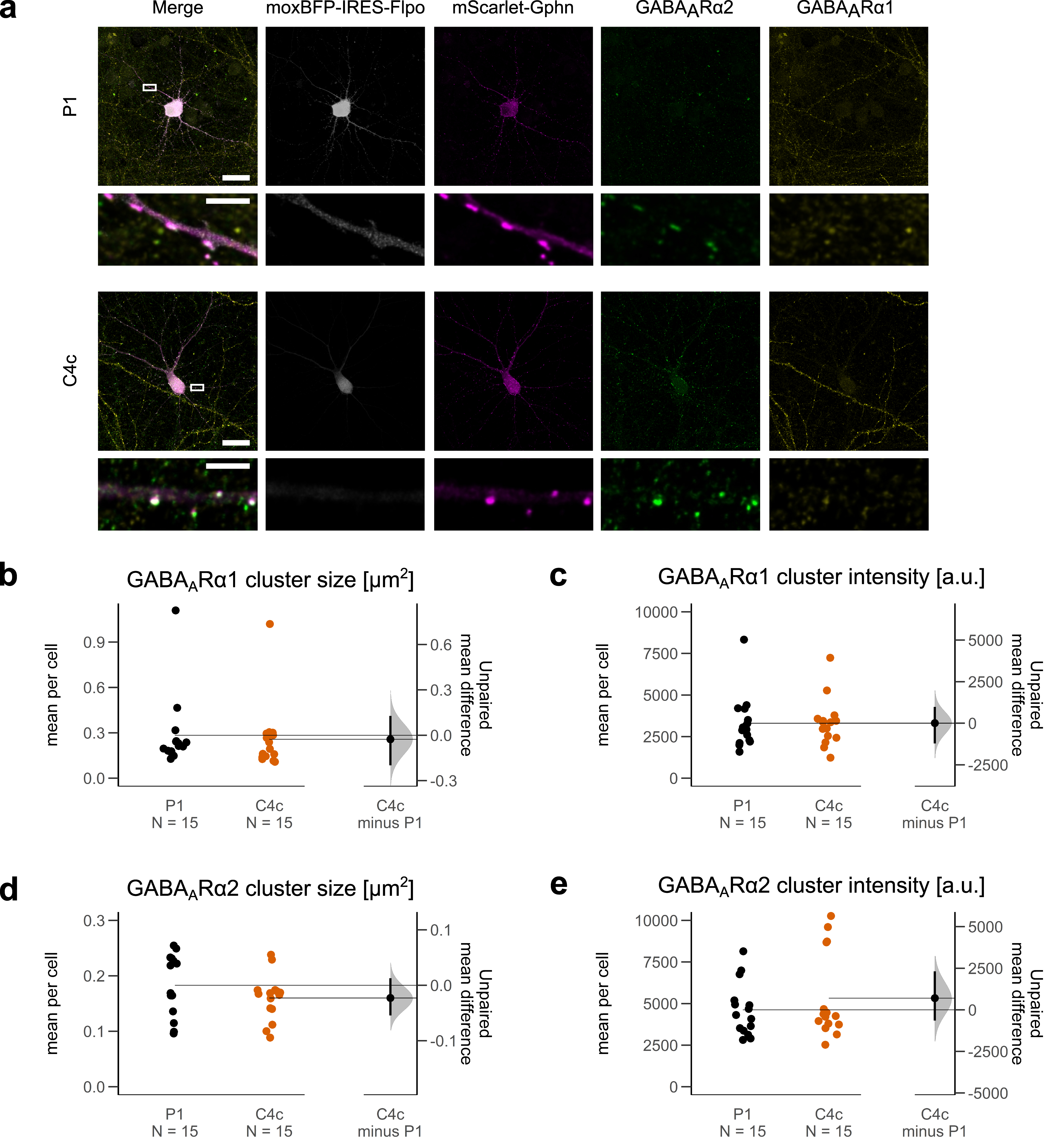

Supplement: Extended Data Figure 8-1 — Expression and analysis of mScarlet-tagged Gphn P1 and C4c isoforms in CamKIIα-expressing murine hippocampal neurons stained for GABAARα1 and GABAARα2. a, Hippocampal cultures were transfected after 8 DIV with moxBFP-IRES-Flpo under the control of the CamKIIα promoter. After 9 DIV, cultures were transduced with adeno-associated virus (AAV) 2/1 carrying Flp dependent mScarlet-tagged Gphn P1 or C4c variants as transgene. Cells were immunostained for GABAARα1 and GABAARα2 after 15 DIV. Representative confocal images (with adaptive image reconstruction) of neurons expressing moxBFP-IRES-Flpo and mScarlet-tagged Gphn. Scale bars: 25 μm and 2.5 μm in insets. b–e, Estimation plots of GABAARα1 and GABAARα2 cluster sizes and fluorescence intensities at P1 and C4c clusters. b, GABAARα1 cluster sizes (average per cell) are not different at P1 and C4c clusters; unpaired two-samples Wilcoxon test, W = 128, p = 0.539. c, GABAARα1 cluster intensities (average per cell) are not different at P1 and C4c clusters; unpaired two-samples Wilcoxon test, W = 105, p = 0.775. d, GABAARα2 cluster sizes (average per cell) are not different at P1 and C4c clusters; unpaired two-sample t test t(28) = 1.296, p = 0.206. e, GABAARα2 cluster intensities (average per cell) are not different at P1 and C4c clusters; unpaired two-samples Wilcoxon test, W = 102, p = 0.683. Each data point represents the mean for an individual neuron. The filled curves indicate the resampled Δ distribution (5.000 bootstrap samples) derived from the observed data. The Δ is indicated by the black circle, which is horizontally aligned with the mean of the test group. The 95% confidence interval of the mean difference is illustrated by the black vertical line. Download Figure 8-1, TIF file. [file enu-eN-NWR-0102-22-s07.tif]
